# Supplementary material for: Deciphering triterpenoid saponin biosynthesis by leveraging transcriptome response to methyl jasmonate elicitation in Saponaria vaccaria
Source: Nat Commun. 2023 Nov 4;14:7101. doi: 10.1038/s41467-023-42877-0 (PMC10625584; doi:10.1038/s41467-023-42877-0)
Supplement: Supplementary file 1 — Supplementary Information [file 41467_2023_42877_MOESM1_ESM.pdf]

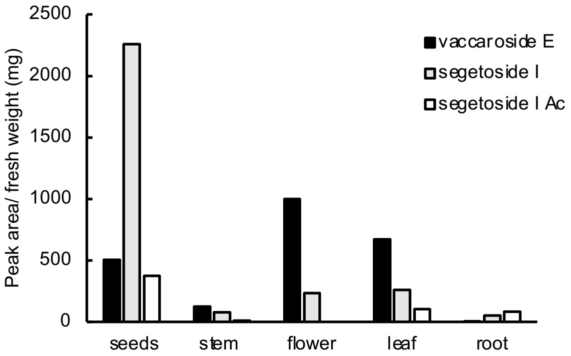

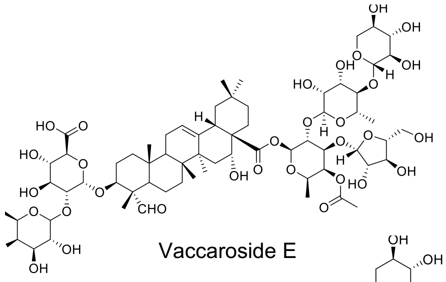

Vaccaroside E

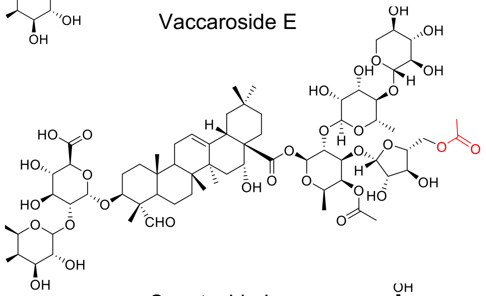

Segetoside I

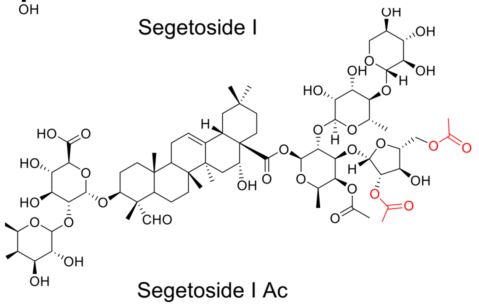

Segetoside I Ac

Supplementary Figure 1. Detection of target mass of vaccaroside E, segetoside I and segetoside I Ac by LC-MS in different tissues of *S. vaccaria*. Source data are provided as a Source Data file.

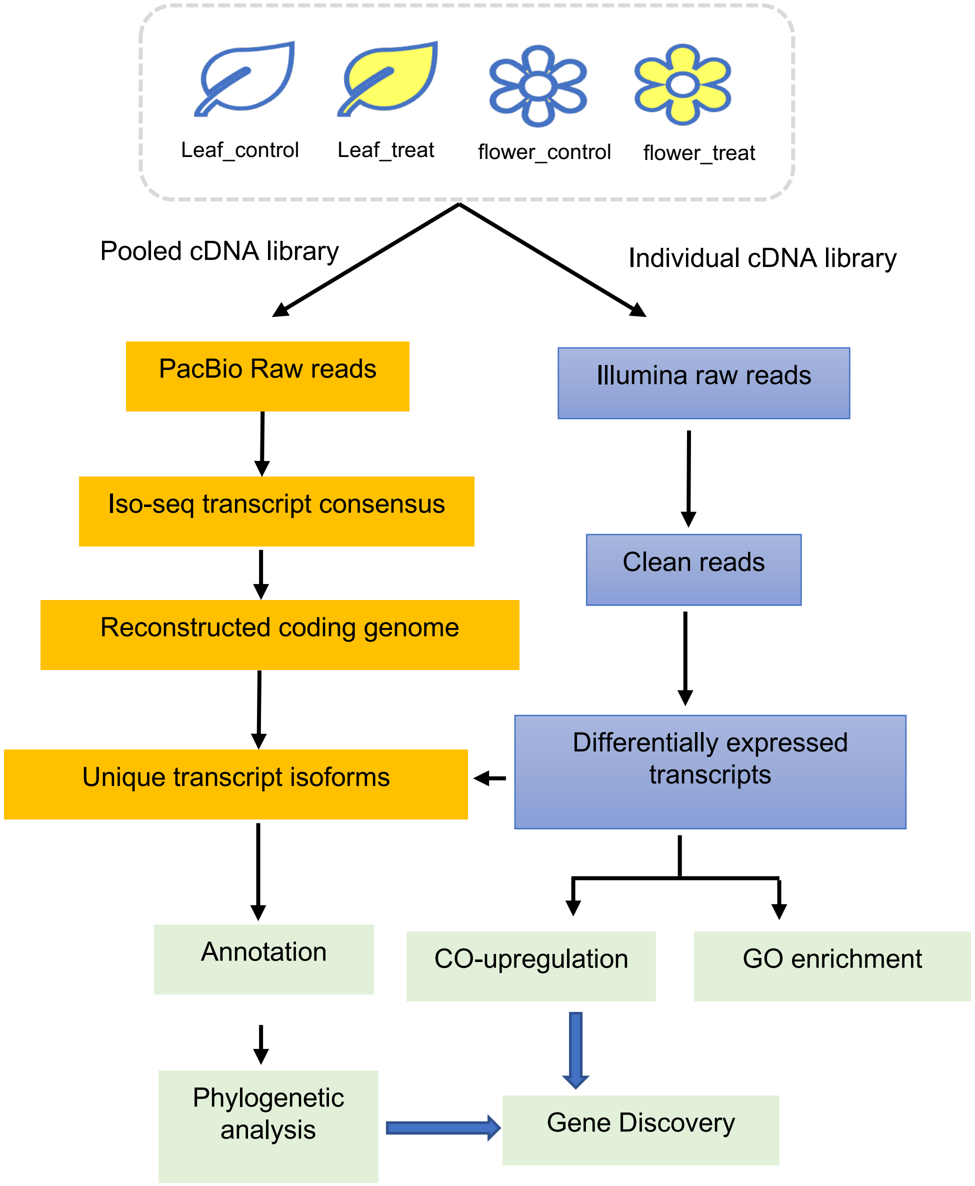

Supplementary Figure 2. Schematic representation of pipeline used for hybrid sequencing data analysis for discovery of genes in saponin biosynthesis pathway in *S. vaccaria*.

a

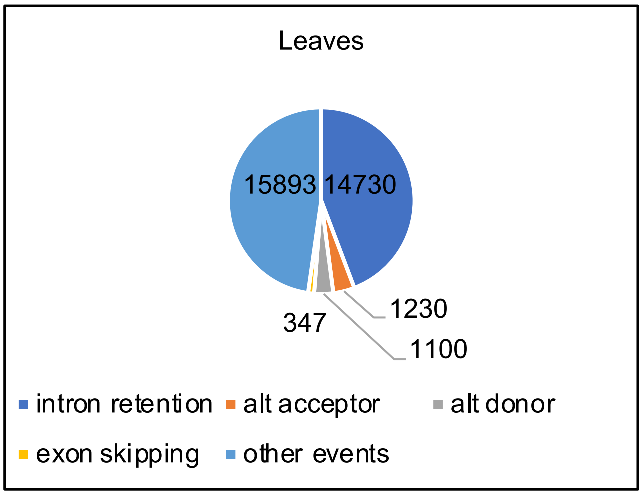

b

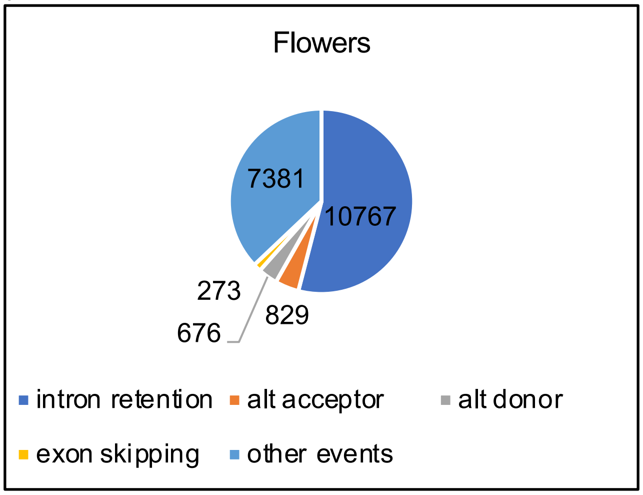

Supplementary Figure 3. Alternative splicing events detected in leaves (**a**) and flowers (**b**) of *S. vaccaria*. Source data are provided as a Source Data file.

a

b

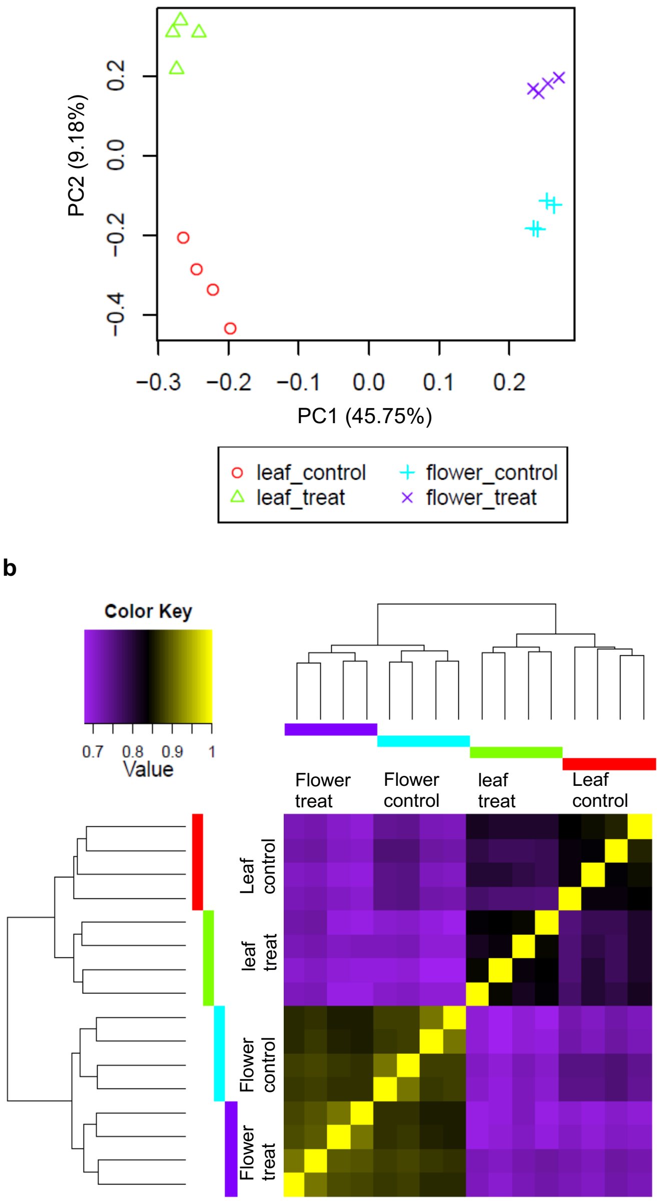

Supplementary Figure 4. Sample replicates correlation analysis of transcript expression profiles. (**a**) PCA plot of transcript counts of biological replicates across all samples. (**b**) Pearson correlation heatmap of transcript counts of biological replicates across all samples.

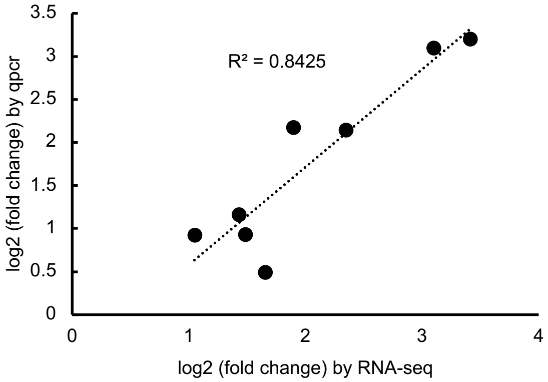

Supplementary Figure 5. Correlation of gene expression fold change between qPCR and RNA-seq (R^2 = 0.84) based on leaf and flower expression of *3-hydroxy-3-methylglutaryl coenzyme A reductase* (*HMGR*), *mevalonate diphosphate decarboxylase* (*MVD*), *squalene synthase* (*SQS*), and *β-amyrin synthase* (*βAS*), which are genes involved in saponin biosynthesis. Source data are provided as a Source Data file.

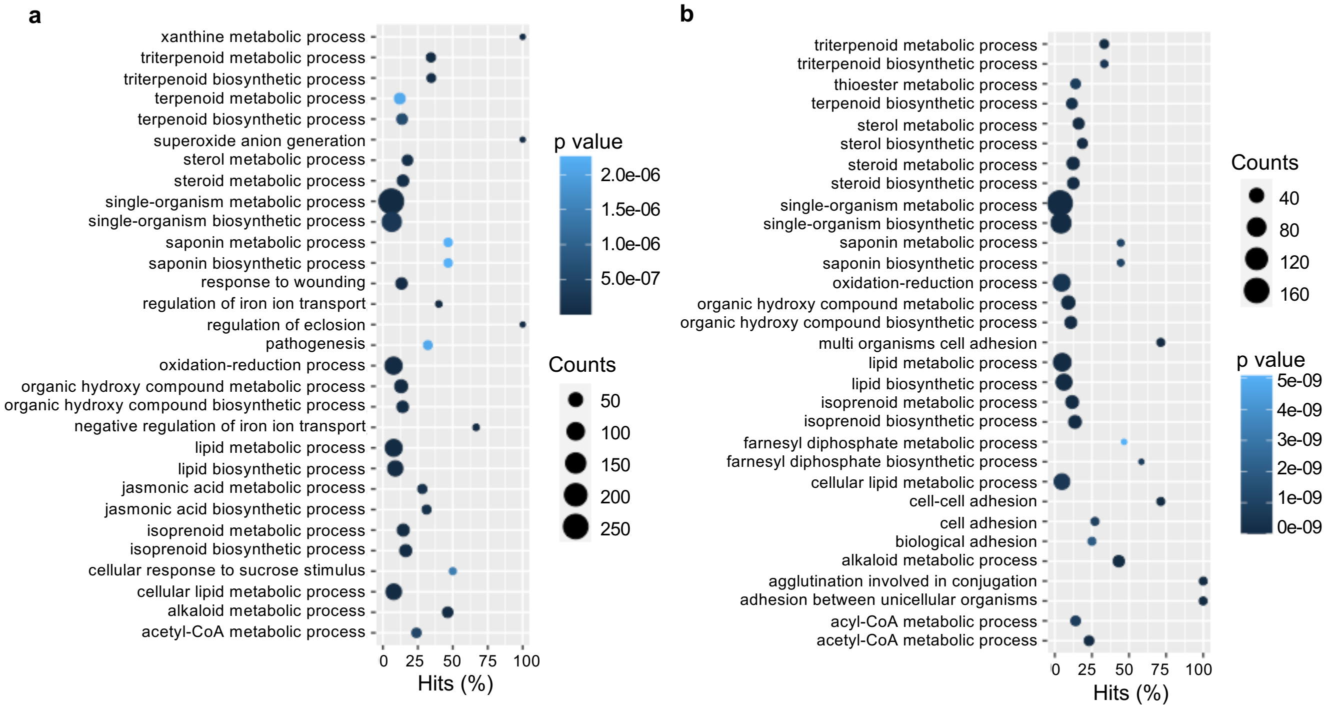

Supplementary Figure 6. GO-enrichment analysis of upregulated genes in leaves (a) and flowers (b) of *S. vaccaria* under MeJA treatment. Y-axis: enriched GO term; x-axis: the percentile of the number of observed genes divided by the number of genes from each GO category.

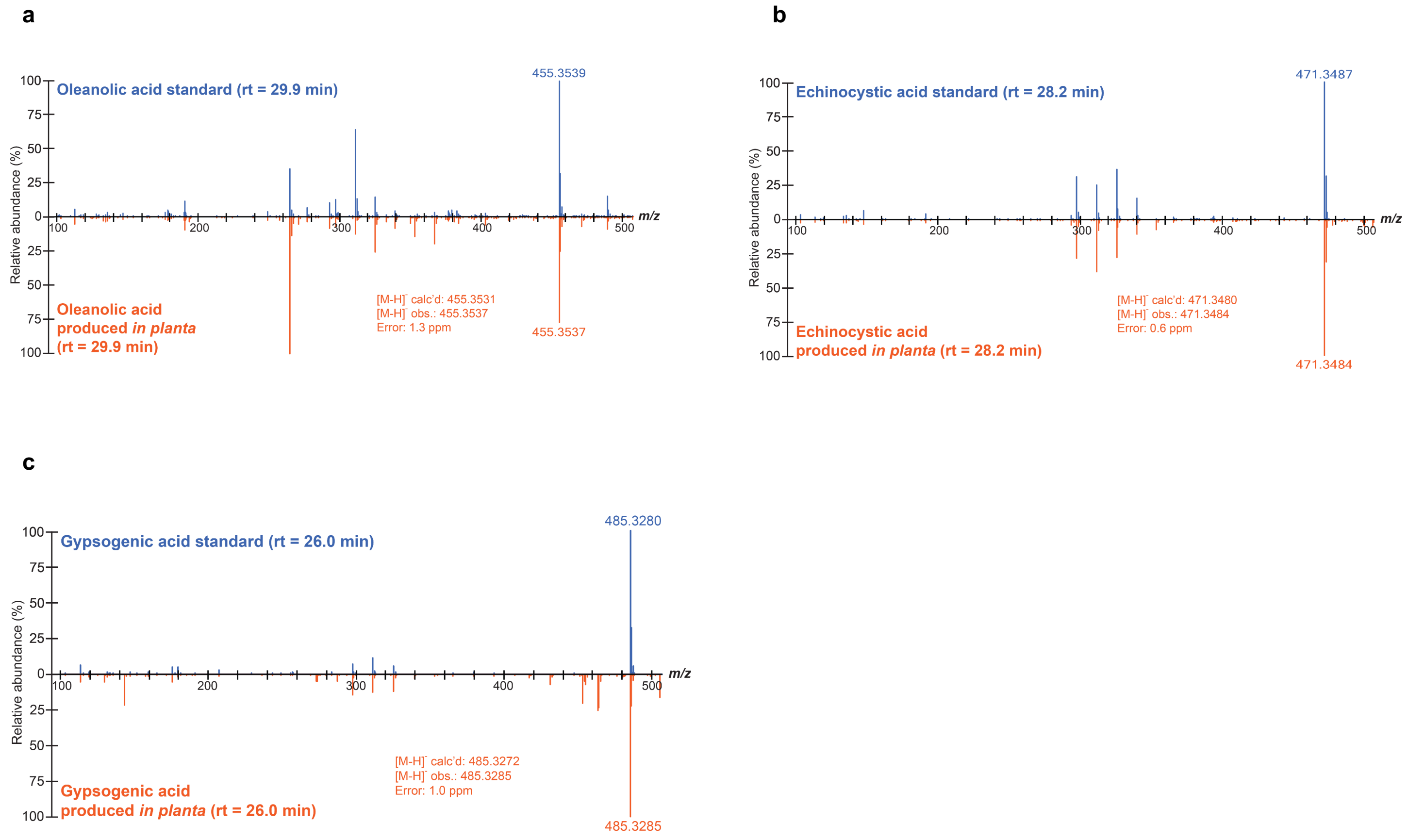

Supplementary Figure 7: High resolution MS of oleanolic acid (**a**), echinocystic acid (**b**), and gypsogenic acid (**c**) produced in *Nicotiana benthamiana* transiently expressing *SvβAS* and CYP candidates as indicated. The figure corresponds to Figure 3 in the main text.

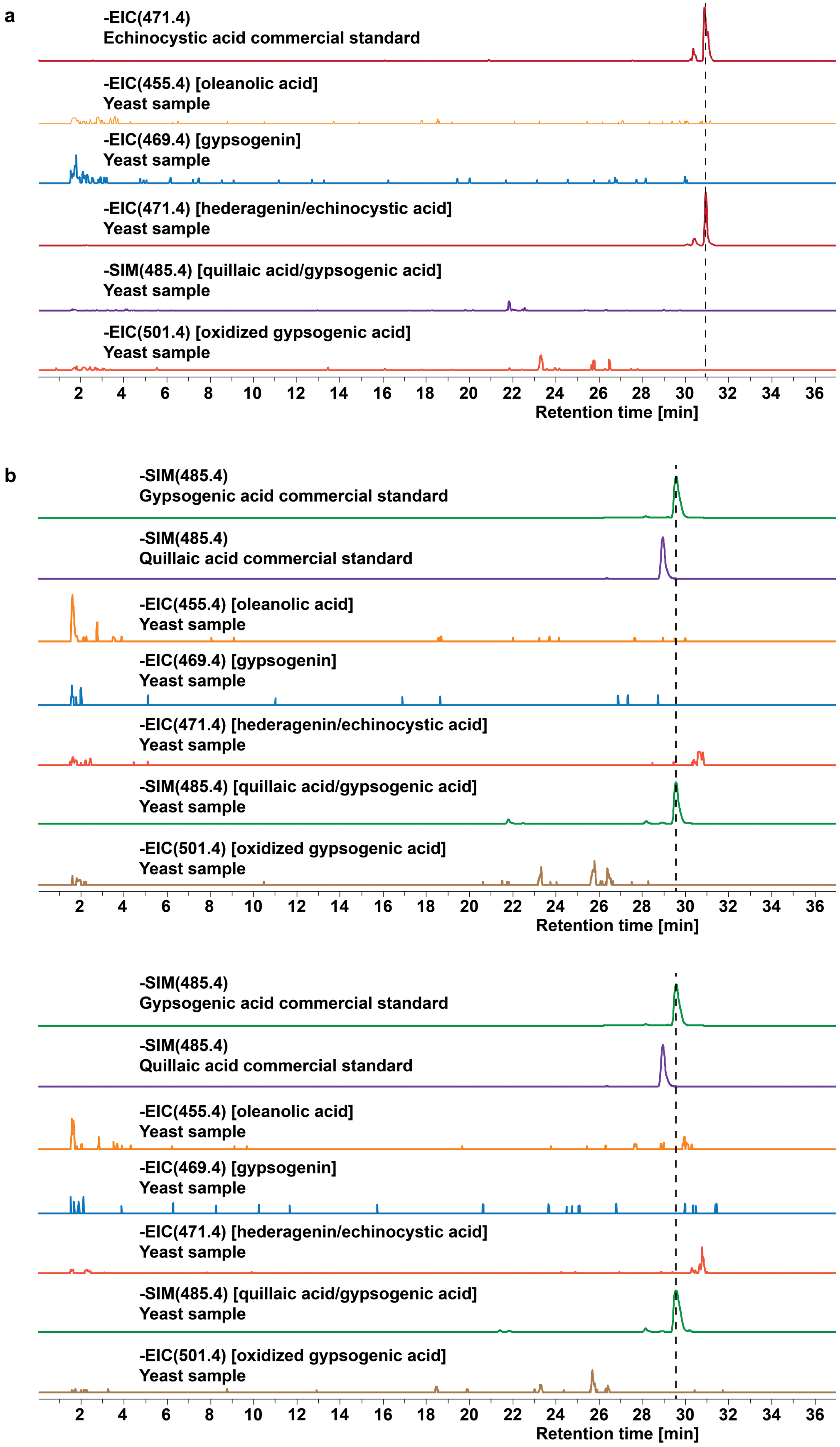

Supplementary Figure 8. Functional test of SvC28, SvC16, and C23 oxidases in β-amyrin producing yeast. (a) Yeast expressing the *SvC16 oxidase* in addition *to SvC28 oxidase* and *AtATR1* on plasmid produce only echinocystic acid. Yeast expressing the *SvC23 oxidase-1* (**b**) or *SvC23 oxidase-2* (**c**) in addition to only *SvC28 oxidase* and *AtATR1* on plasmid produce exclusively gypsogenic acid.

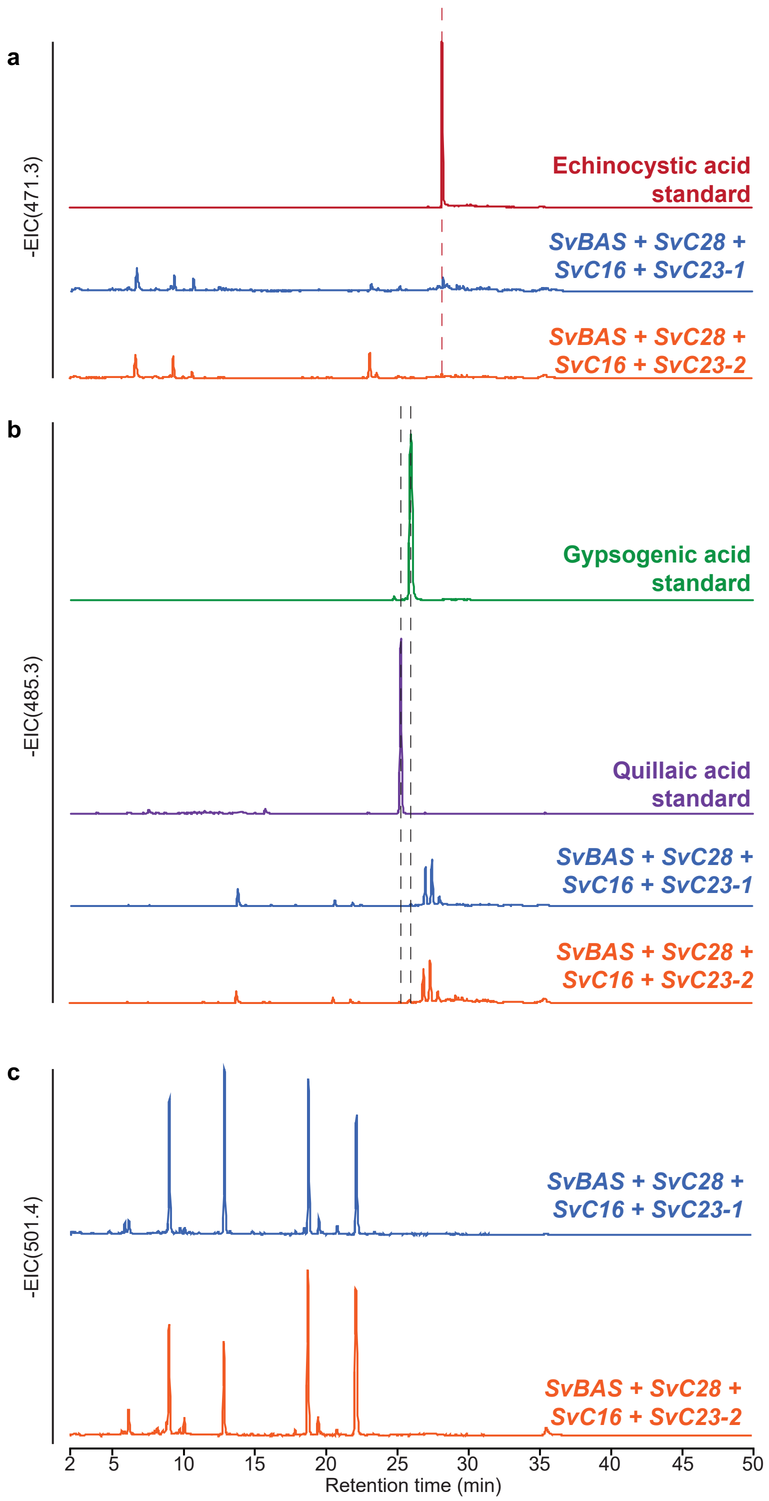

Supplementary Figure 9. Combined expression of *SvC28*, *SvC16*, and *SvC23 oxidases* in *N. benthamiana*. EIC of echinocystic acid (**a**), quillaic acid / gypsogenic acid (**b**), and oxidized gypsogenic acid (**c**) in leaves expressing *SvβAS*, *SvC28*, and *SvC16* together with *SvC23-1* or *SvC23-2*.

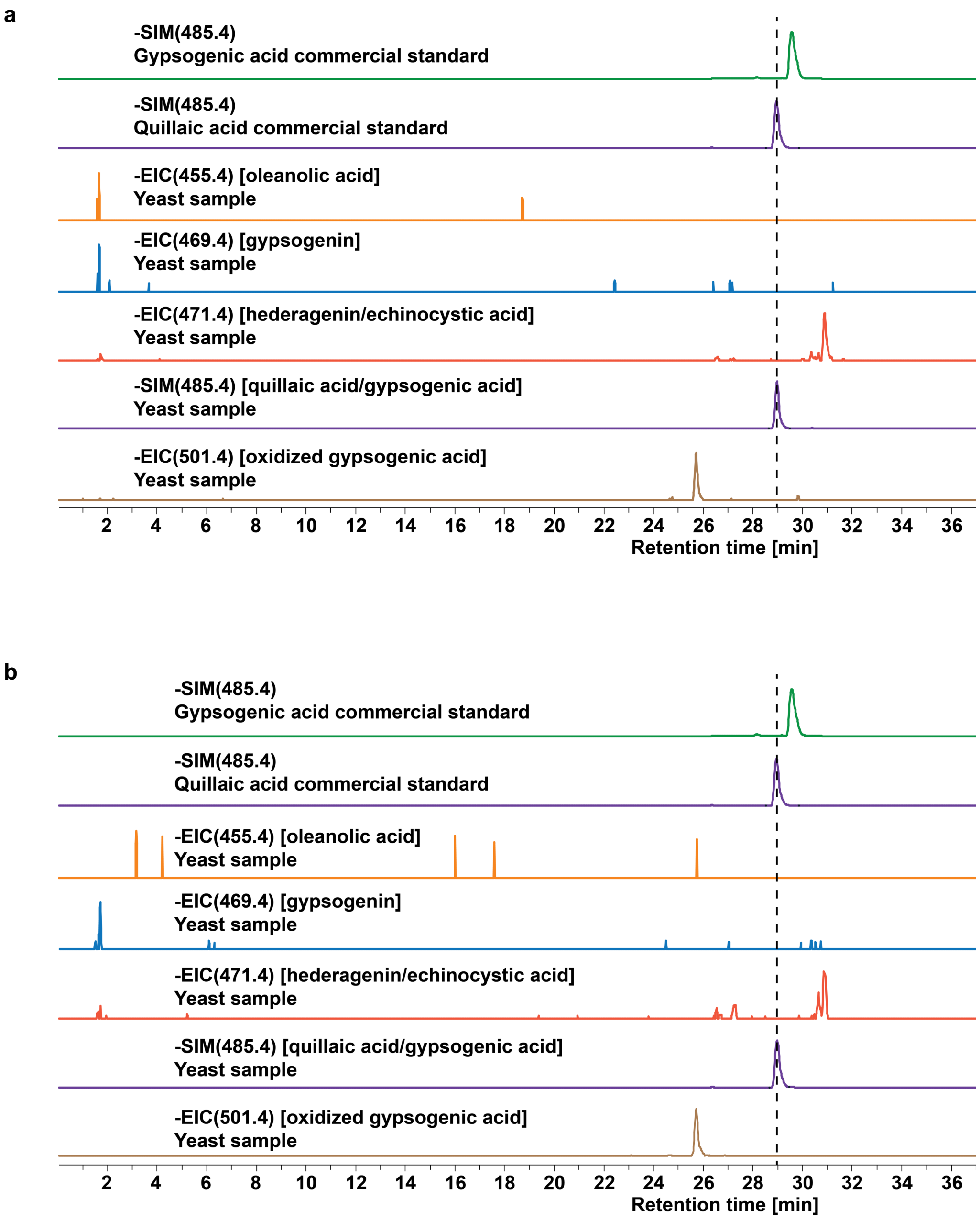

Supplementary Figure 10. Yeast expressing the *SvC23 oxidase-1* (**a**) or *SvC23 oxidase-2* (**b**) in addition to *SvC28* and *SvC16* oxidases and *AtATR1* on plasmid produce predominantly quillaic acid with smaller amounts of oxidized gypsogenic acid and echinocystic acid detectable.

a

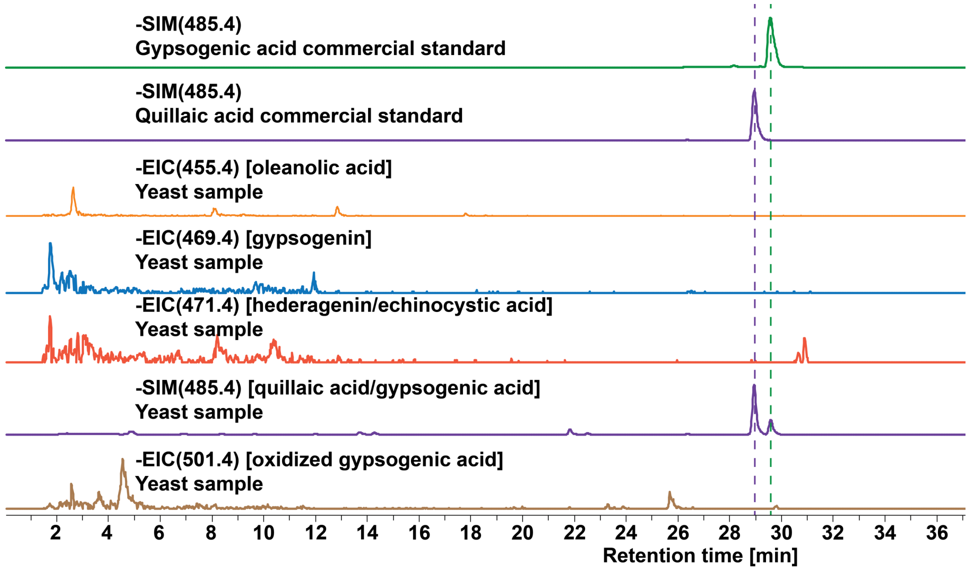

b

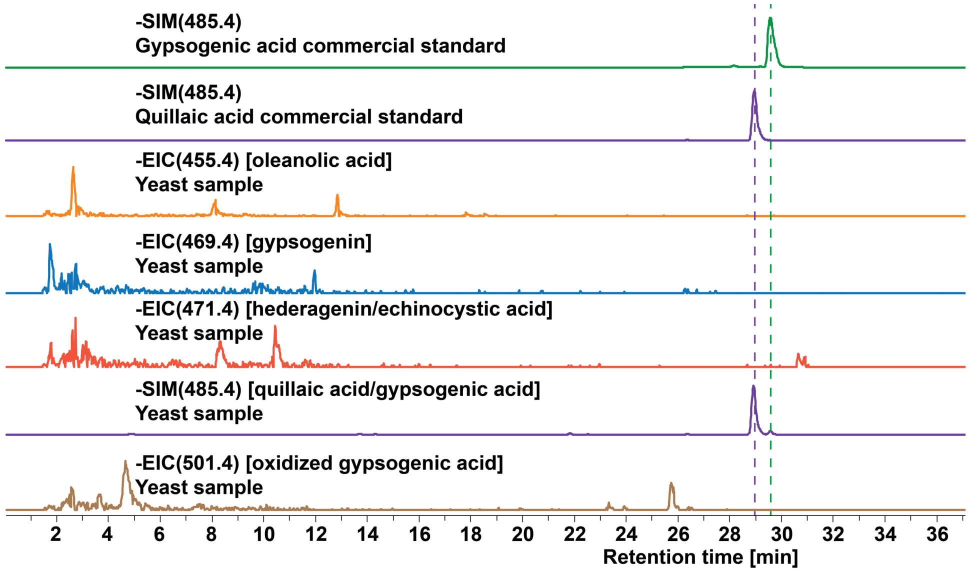

Supplementary Figure 11. Yeast expressing the *SvC23 oxidase-1* (**a**) or *SvC23 oxidase-2* (**b**) in addition to *SvC28* and *SvC16* oxidases and *AtATR1* integrated into the yeast genome produces predominantly quillaic acid as well as a small amount of gypsogenic acid.

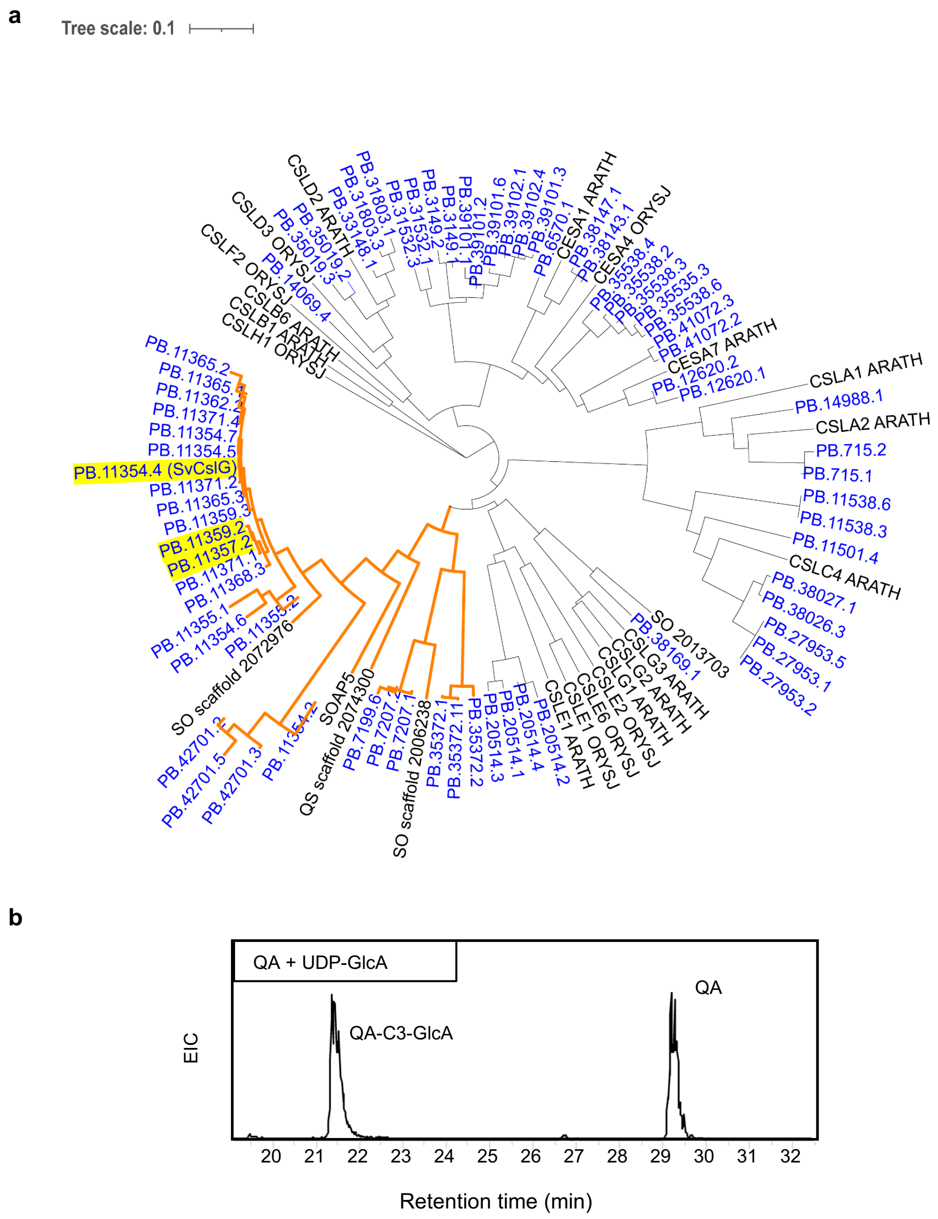

Supplementary Figure 12. Identification of quillaic acid-C3-glucuronosyltransferase. (**a**) A neighbor-joining tree (1,000 bootstrap replicates) of Csl genes of *S.vaccaria* and other plants. Gene names in blue represent *S.vaccaria* Csl candidates. Yellow background highlight indicates co-expression with β amyrin synthase. Only the transcript of *SvCslG* (PB.11354.4) encodes a full-length protein among co-expressed genes. Genes from *Arabidopsis thaliana* (ARATH) and *Oryza Sativa subsp. Japonica* (ORYSJ) are designated as CSL class and species name. Accession numbers of Csl proteins are in Supplementary Table 2 and sequences of *S. vaccaria* proteins in the tree are in Supplementary Data 1. (**b**) EIC of quillaic acid and quillaic acid 3-O-glucuronide (QA-3-GlcA) of SvCslG in vitro enzyme assay with QA and UDP-GlcA as substrates.

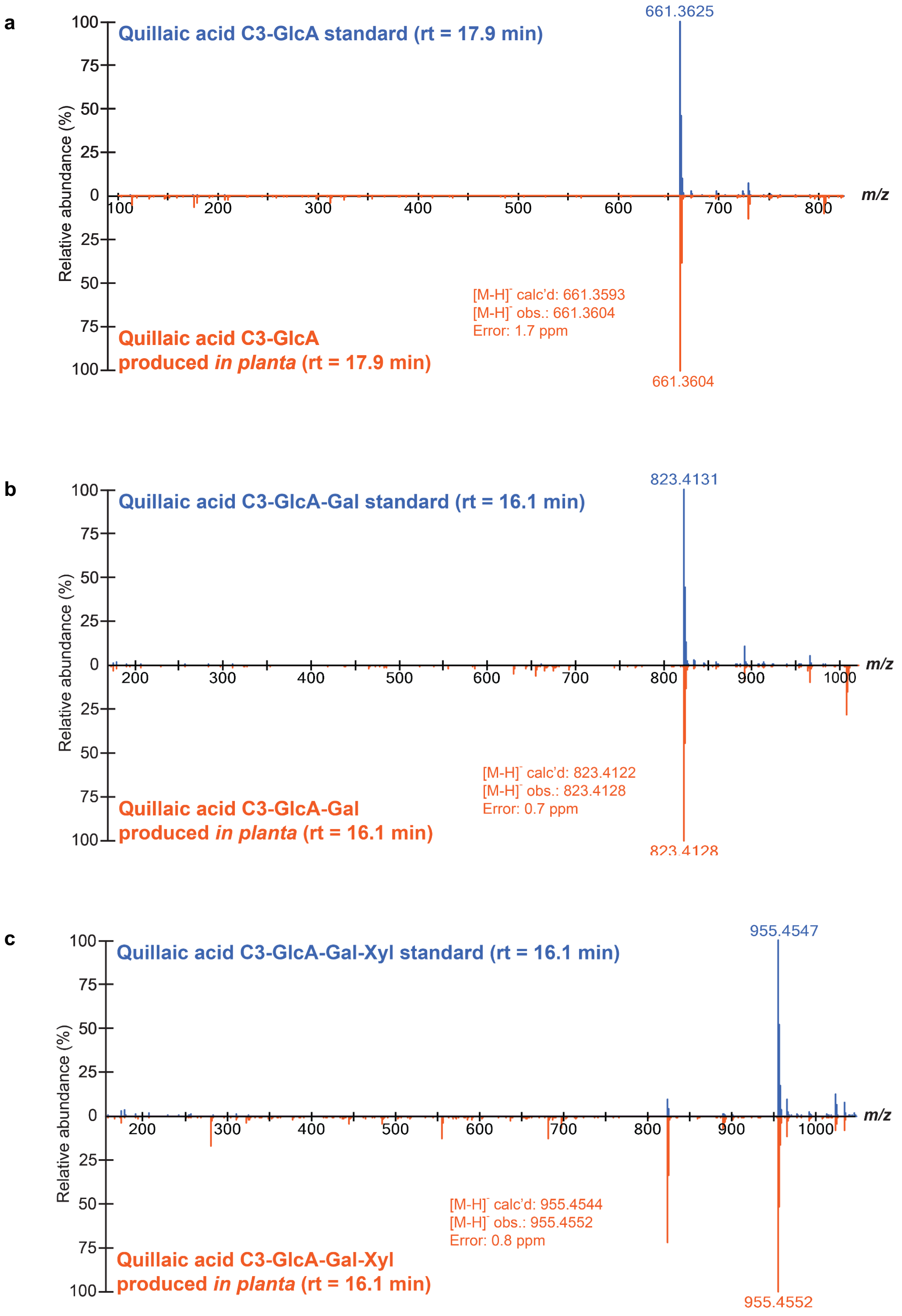

Supplementary Figure 13. High resolution MS of QA-C3-GlcA, QA-C3-GlcA-Gal and QA-C3-GlcA-Gal-Xyl from plants transiently expressing (**a**) *SvCslG,* **(b)** *SvCslG* + *SvGalT*, and (**c**) *SvCslG* + *SvGalT* + *SvC3XylT* and infiltrated with QA solution. The figure shows mass spectra corresponding to Figure 5 in the main text.

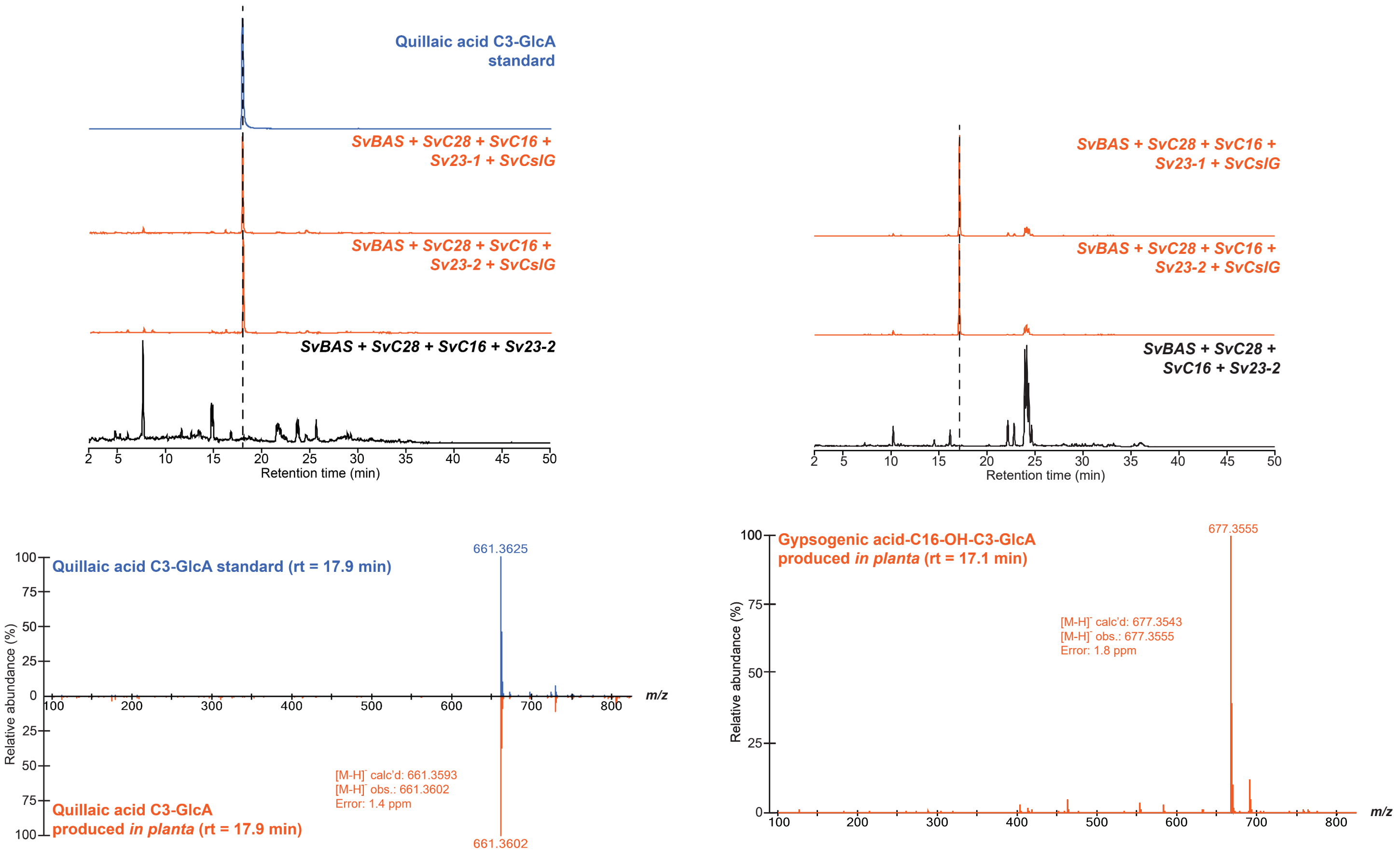

Supplementary Figure 14. EIC and high resolution MS of QA-C3-GlcA (**a**) and GA-C16-OH-C3-GlcA (**b**) in *N. benthamiana* expressing *SvβAS*, *SvC28*, *SvC16*, and *SvC23-1/-2* together with *SvCslG* or p19 strain. No standard is available for GA-C16-OH-C3-GlcA. The data corresponds to Figure 6 in the main.

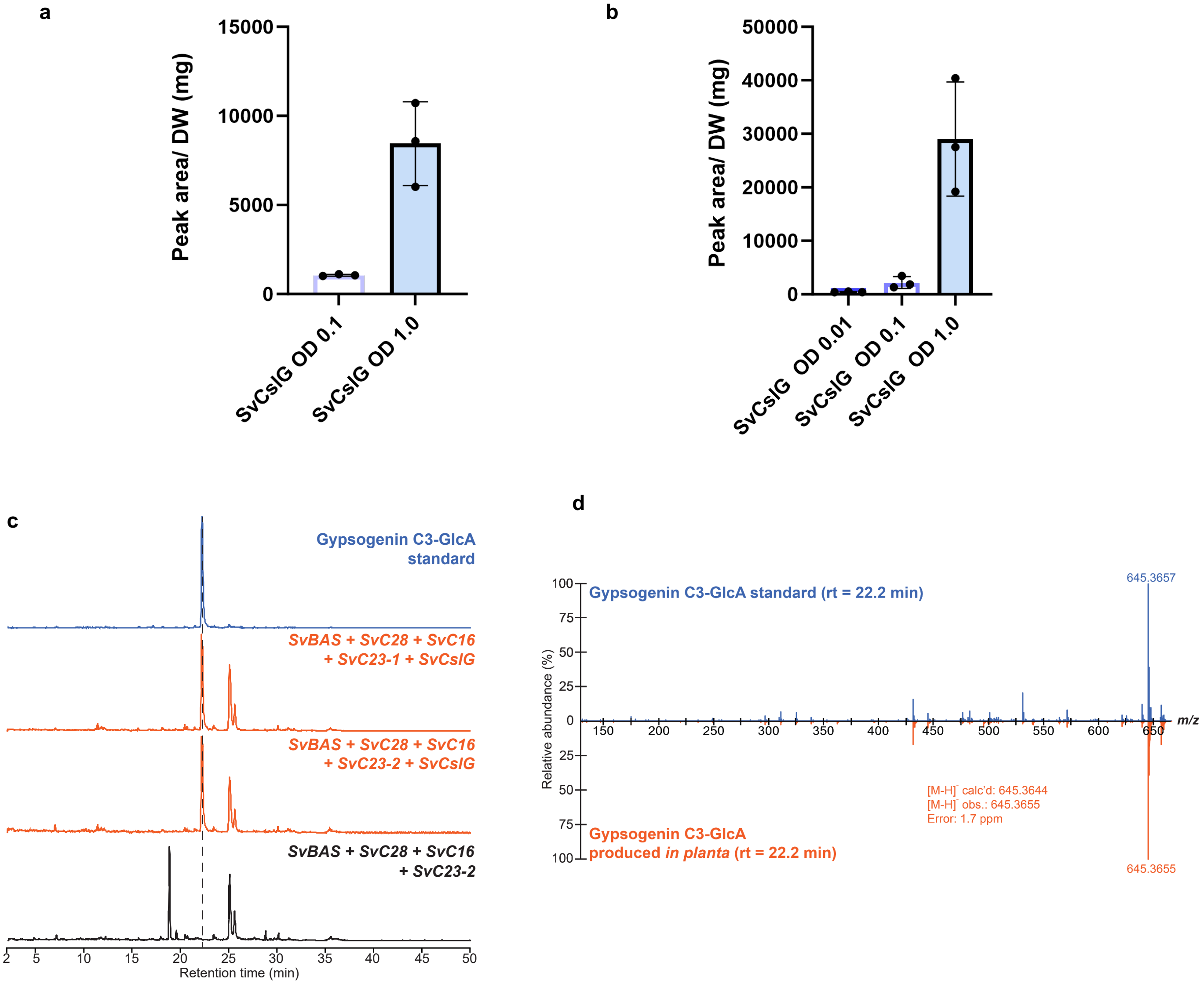

Supplementary Figure 15. SvCslG increases the production of gypsogenin-C3-GlcA. Different OD levels of *Agrobacterium tumefaciens* harboring *SvCslG* were individually co-infiltrated with *A. tumefaciens* harboring *SvβAS*, *SvC28*, *SvC16* and *SvC23-1* (**a**) and *SvC23-2* (**b**). Error bars indicate mean ± SD (n=3 biologically independent samples). Source data and test statistics are provided as a Source Data file. (**c**) EIC for the different enzyme combinations. (**d**) Mass spectrum of gypsogenin C3-GlcA.

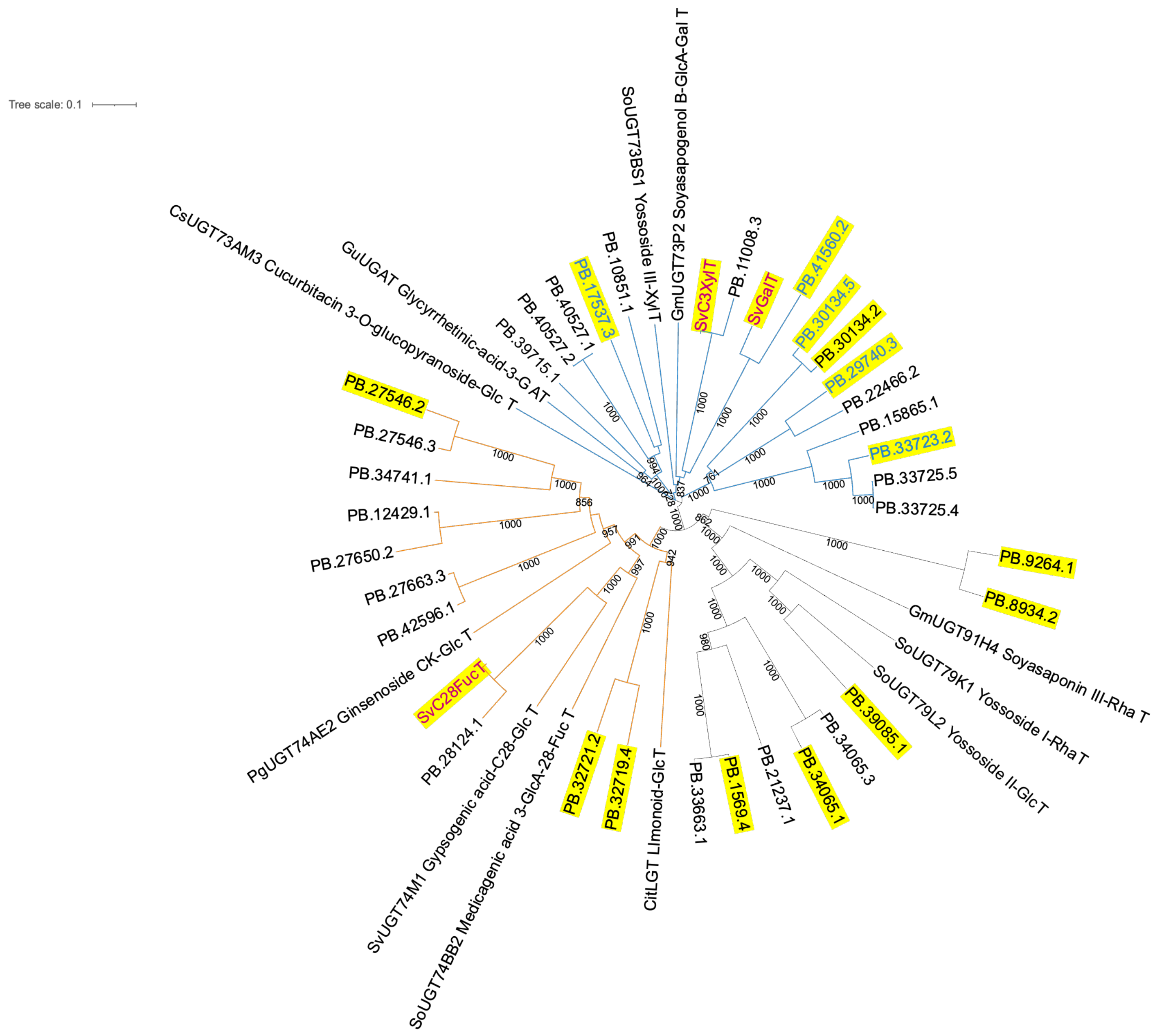

Supplementary Figure 16. Neighbor-joining tree (1,000 bootstrap replicates) of SvUGTs and other characterized GT1s glycosylating triterpenoids. The identified SvGalT, SvC3XylT, and SvC28FucT are shown in red letter. Five candidates of SvGalT that have been functionally tested are shown in blue. Yellow highlight indicates co-expression with *SvβAS*. Genes from other plants are designated as name followed by function description. Sequences of *S. vaccaria* proteins in the tree are in Supplementary Data 1 and accession numbers of GT1s from other species are in Supplementary Table 2 and

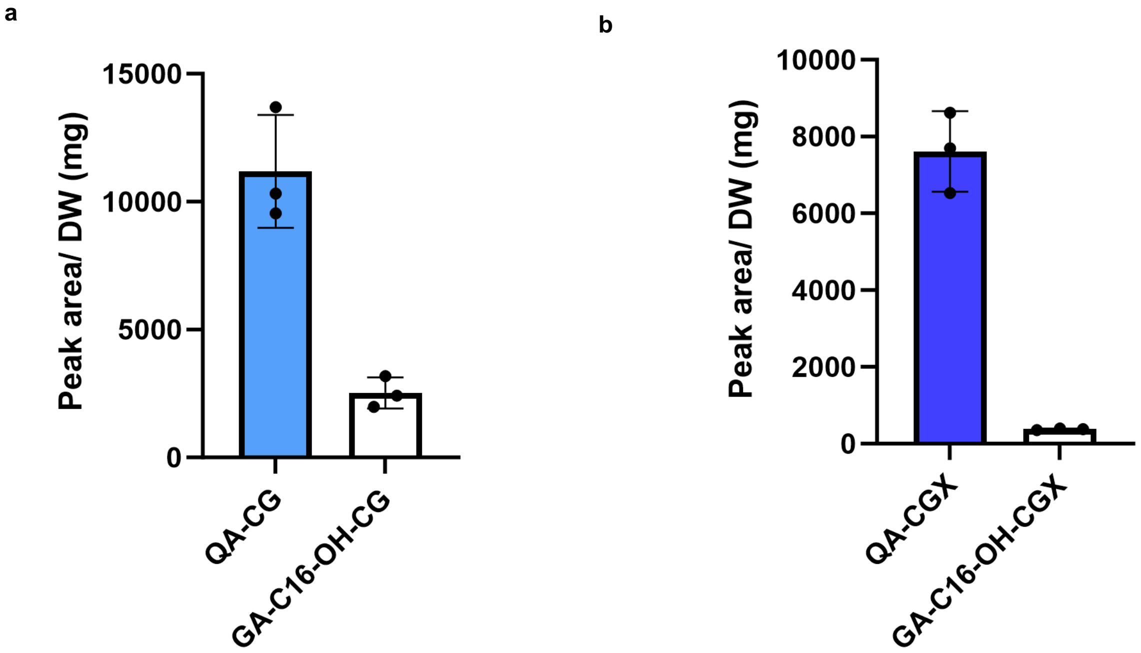

Supplementary Figure 17. SvGalT and SvC3XylT further boost the C3 glycosylation of C23 aldehyde. (**a**) formation of QA-C3-GlcA-Gal and GA-C16-OH-C3-GlcA-Gal in *N. benthamiana* expressing *SvβAS*, *SvC28*, *SvC16*, *SvC23-1*, and *SvGalT*. (**b**) Production of QA-C3-GlcA-Gal-Xyl and GA-C16-OH-C3-GlcA-Gal-Xyl in *N. benthamiana* expressing *SvβAS*, *SvC28*, *SvC16*, *SvC23-1*, *SvGalT*, and *SvXylT*. Error bars indicate mean ± SD (n=3 biologically independent samples). Source data and test statistics are provided as a Source Data file.

```
Sv46DH/1-380     1 MRLIFMFFGGKAGKMVDYTPKNILITGAAGFIASHVANRLVRNYPEYNIVVLDKLDYCSNLKNLIPSRSSSKFKF  75
SvRHM/1-651      1 --------------MANYIPKNILITGAAGFIASHVANRLVRDYPDYKIIVLDKLDYCSNLKNLNPSRSSPHFKF  61
AtRHM1/1-669     1 --------------MASYTPKNILITGAAGFIASHVANRLIRSYPDYKIVVLDKLDYCSNLKNLNPSKHSPNFKF  61

Sv46DH/1-380    76 IKGDIASADLVNFILTTEAIDTIMHFAAQTHVDNSFGNSFEFTKNNIYGTHVLLEACKVTGKQIKRFIHVSTDEV 150
SvRHM/1-651     62 IKGDIASADLVNYILTTEAIDTIMHFAAQTHVDNSFGNSFEFTKNNIYGTHVLLEACKLTGPQIKRFIHVSTDEV 136
AtRHM1/1-669    62 VKGDIASADLVNHLLITEGIDTIMHFAAQTHVDNSFGNSFEFTKNNIYGTHVLLEACKVTG-QIRRFIHVSTDEV 135

Sv46DH/1-380   151 YGETDEDAAVGNHETSQLLPTNPYSATKAGAEMLVMAYGRSYGLPVITTRGNNVYGPNQFPEKMIPKFILLAMRG 225
SvRHM/1-651    137 YGETDEDAAIGNHEASQLLPTNPYSATKAGAEMLVMAYGRSYGLPVITTRGNNVYGPNQFPEKMIPKFILLAMKG 211
AtRHM1/1-669   136 YGETDEDALVGNHEASQLLPTNPYSATKAGAEMLVMAYGRSYGLPVITTRGNNVYGPNQFPEKLIPKFILLAMRG 210

Sv46DH/1-380   226 LPLPIHGDGSNVRSYLYCEDVAEAFETILHKGEVGHVYNIGTLKERRVIDVAKEICTLFNRNPDETIKFVENRPF 300
SvRHM/1-651    212 LPLPIHGDGSNVRSYLYCEDVAEAFETILHKGEIGHVYNIGTLKERTVLDVAKEICNLFSRNPDETIKFVENRPF 286
AtRHM1/1-669   211 QVLPIHGDGSNVRSYLYCEDVAEAFEVVLHKGEVGHVYNIGTKKERRVNDVAKDICKLFNMDPEANIKFVDNRPF 285

Sv46DH/1-380   301 NDQRYFLDDQKLKKLGWSERTSWKEGLIKTMEWYVKNPDWWGDVSGALVPHPRMLMMPGG--TDGRINGNGTSIT 373
SvRHM/1-651    287 NDQRYFLDDQKLKRLGWSERTNWKEGLLKTMEWYVRNPDWWGDVSGALVPHPRMLMMPGG--ADGRVNGNETTFP 359
AtRHM1/1-669   286 NDQRYFLDDQKLKKLGWSERTTWEEGLKKTMDWYTQNPEWWGDVSGALLPHPRMLMMPGGRHFDGSEDNSLAATL 360

Sv46DH/1-380   374 ANGNCHS-------------------------------------------------------------------- 380
SvRHM/1-651    360 SQAVKKSSLPTAS-----TVDDSSLKFLIYGKTGWIGDLLGKLLEKQGISYEYGKGRLDDIAQVLDDIRVVKPTH 429
AtRHM1/1-669   361 SEKPSQTHMVVPSQRSNGTPQKPSLKFLIYGKTGWIGGLLGKICDKQGIAYEYGKGRLEDRSSLLQDIQSVKPTH 435

Sv46DH/1-380       ---------------------------------------------------------------------------
SvRHM/1-651    430 VFDAAGMTGRQIAEWCESHKAEAIRANVANTLTLADVCREHGVMLVHYATGCFFGYDDAHPLESCIGFKEDEQPN 504
AtRHM1/1-669   436 VFNSAGVTGRPNVDWCESHKTETIRANVAGTLTLADVCREHGLLMMNFATGCIFEYDDKHPEGSGIGFKEEDTPN 510

Sv46DH/1-380       ---------------------------------------------------------------------------
SvRHM/1-651    505 FTASFYSKTKAIAEEFLKEYENVCILRVQMPVSSDLSNPRNSISNITKYNKVVNITNSMTNLDELLPISIEMAKR 579
AtRHM1/1-669   511 FTGSFYSKTKAMVEELLKEYDNVCTLRVRMPISSDLNNPRNFITKISRYNKVVNIPNSMTVLDELLPISIEMAKR 585

Sv46DH/1-380       ---------------------------------------------------------------------------
SvRHM/1-651    580 KLKGIWNFTNPGVVTNNQILEMYKKYIDPEYKWVNFTSEERSKLVVAPRSNNELDSMKLKNEFRECYPSRSR--- 651
AtRHM1/1-669   586 NLKGIWNFTNPGVVSHNEILEMYRDYINPEFKWANFTLEEQAKVIVAPRSNNEMDASKLKKEFPELLSIKESLIK 660

Sv46DH/1-380       ---------
SvRHM/1-651        ---------
AtRHM1/1-669   661 YAYGPNKKT                                                                   669
```

Supplementary Figure 18. Amino acid Sequence alignment of Sv46DH (PB.29997.1) with AtRHM1 (At1g78570) and putative SvRHM (PB.1557.6).

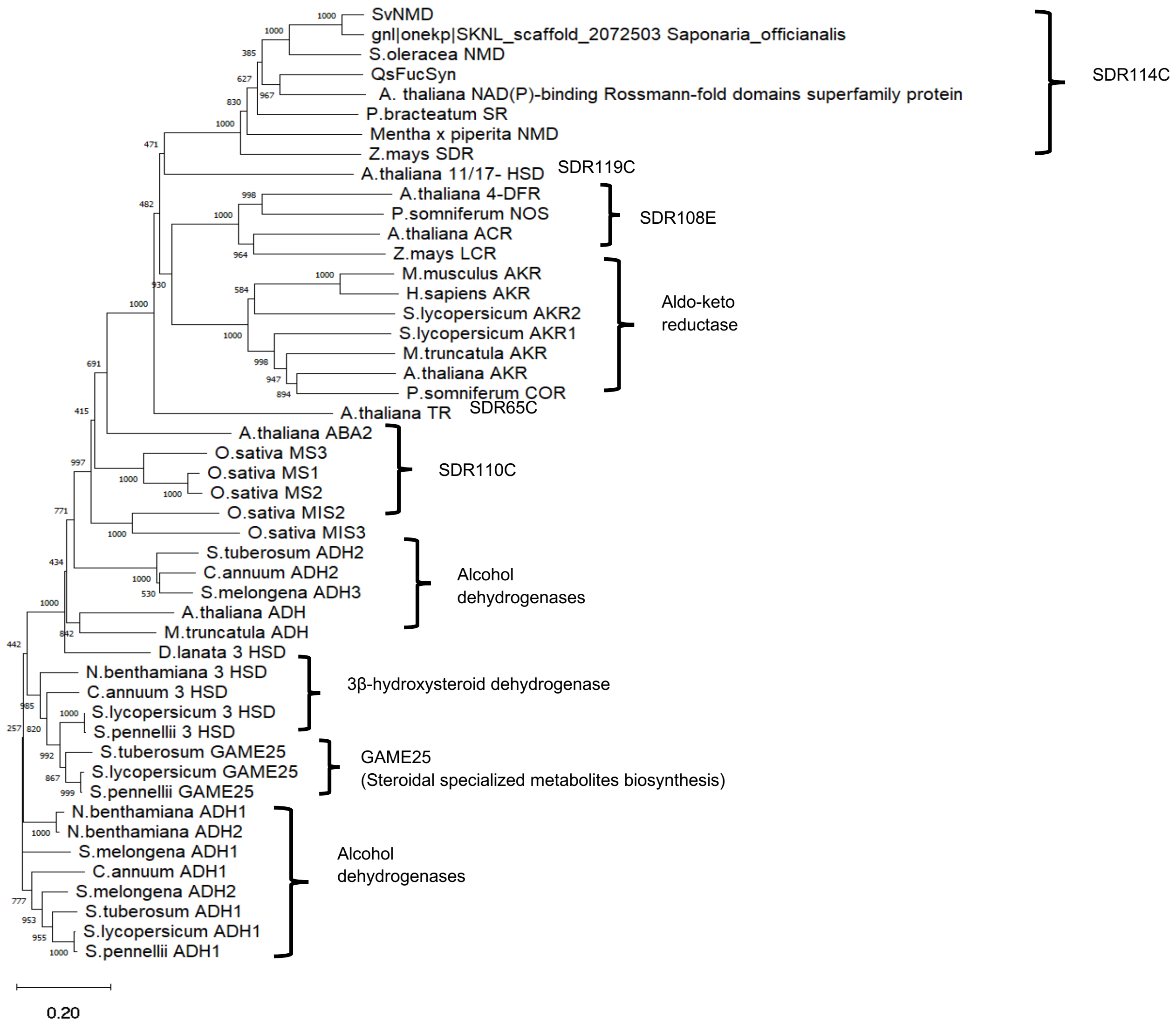

Supplementary Figure 19. Neighbor-joining tree (1,000 bootstrap replicates) of SvNMD and other short-chain dehydrogenases/reductases. The coding sequence of SvNMD is in Supplemental Data 2 and the remaining amino acid sequences are provided in Supplementary Data 3.

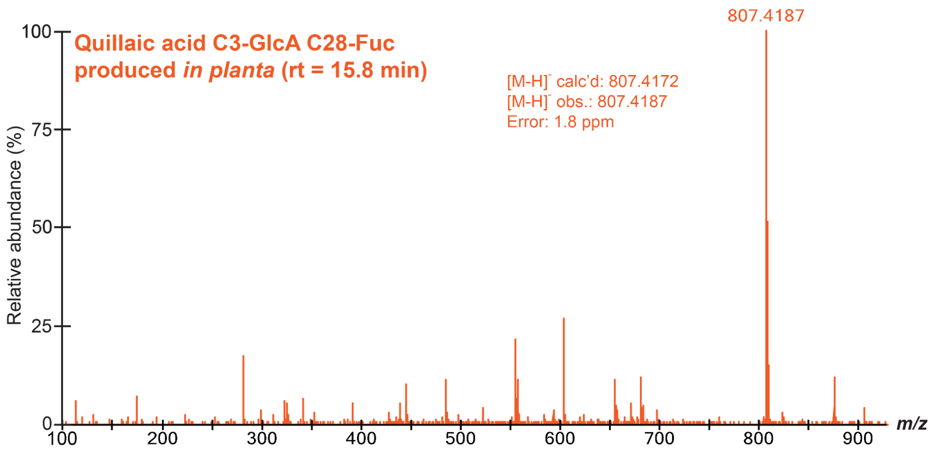

Supplementary Figure 20. Mass spectrum of QA C3-GlcA C28-Fuc produced in planta (no standard is available). The data corresponds to Figure 7 in the main text.

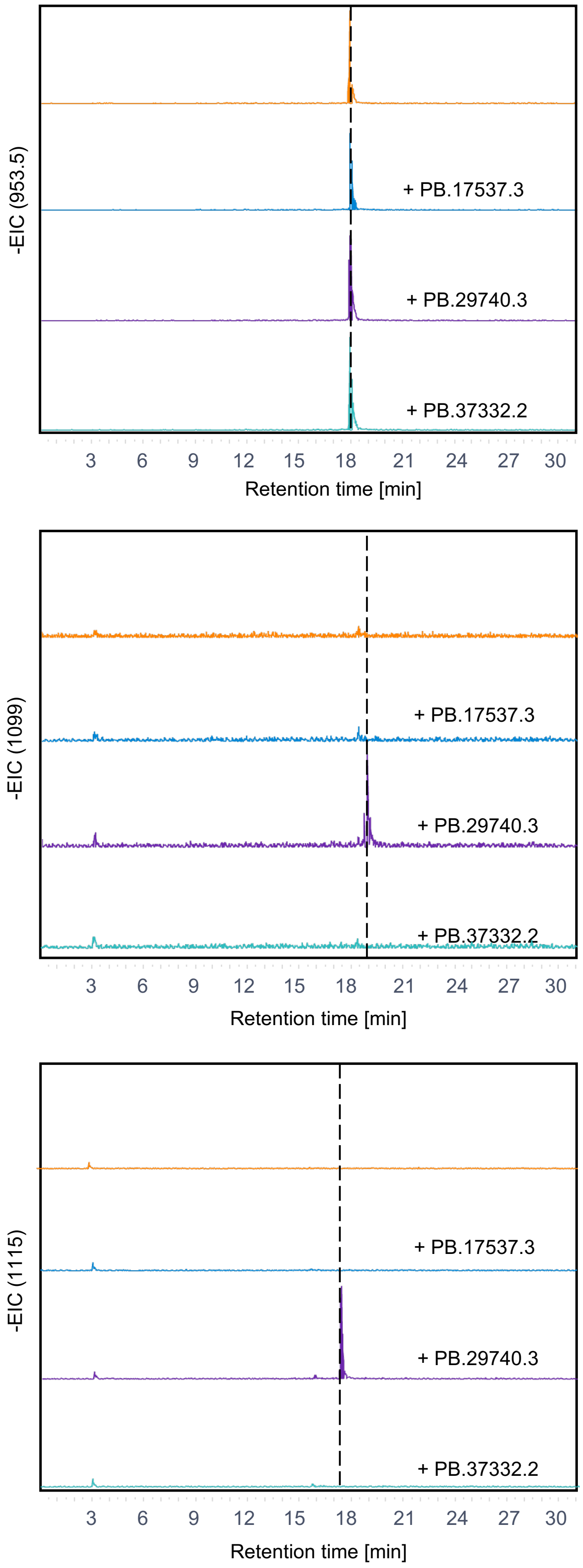

Supplementary Figure 21. EIC of (a) QA-C3-GlcA-C28-Fuc-Rha (*m/z* 953), (b) QA-C3-GlcA-C28-Fuc-Rha-deoxyhexose (*m/z* 1099), and (c) QA-C3-GlcA-C28-Fuc-Rha-hexose (*m/z* 1115) in *N. benthamiana* transiently expressing *SvβAS*, *SvC28*, *QsC16*, *QsC23*, *SvCslG*, *Sv46DH*, *SvNMD*, *SvC28FucT*, and *QsC28RhaT* only (control) or in addition to individual SvC28 GT candidate *PB. 17537.3 (+ PB. 17537.3*), *PB. 29740.3 (+ PB. 29740.3*), or *PB. 33723.2* (+ *PB. 33723.2*).

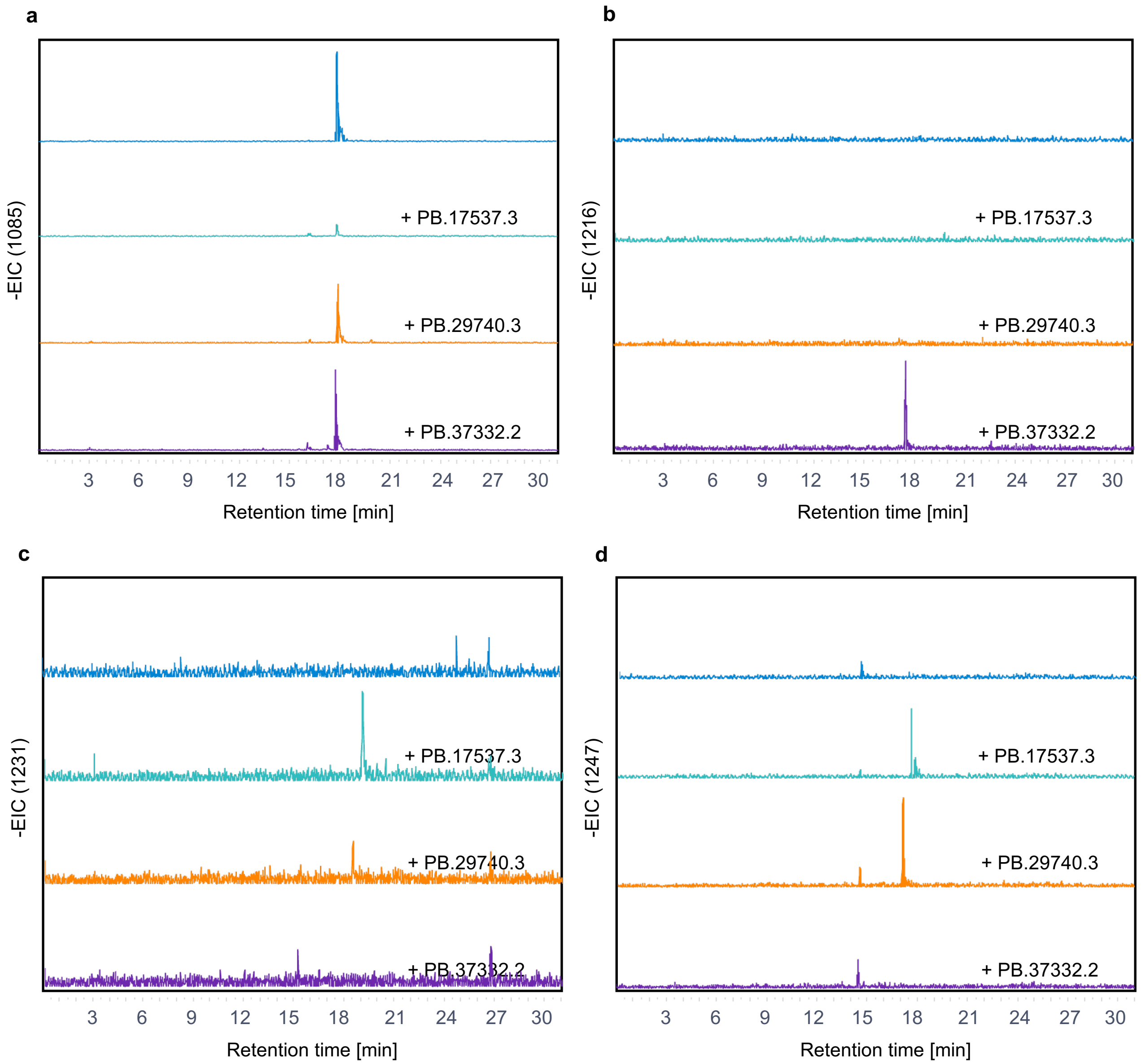

Supplementary Figure 22. EIC of (**a**) QA-C3-GlcA-C28-Fuc-Rha-Xyl (*m/z* 1085), (**b**) QA-C3-GlcA-C28-Fuc-Rha-Xyl-pentose (*m/z* 1217), (**c**) QA-C3-GlcA-C28-Fuc-Rha-Xyl-deoxyhexose (*m/z* 1231), and (**d**) QA-C3-GlcA-C28-Fuc-Rha-Xyl-hexose (*m/z* 1247) in *N. benthamiana* transiently expressing *SvβAS, SvCYPC28, QsC16, QsC23, SvCslG, Sv46DH, SvNMD, SvC28FucT,* and *QsC28RhaT* only (control) or in addition to individual SvC28 GT candidate *PB.17537.3* (+ *PB. 17537.3*), *PB. 29740.3* (+ *PB.29740.3*), or *PB.33723.2* (+ *PB.33723.2*).

Supplementary Table 1. Structure features of major triterpenoid saponins in *S. vaccaria*

Name	Aglycone oxidation	C23 sugar	C3 sugars	C28 sugars
Vaccaroside I[1]	QA (C23CHO, C16OH)		GlcA, Gal	Fuc, Rha, xyl, glc; Fuc
vaccaroside E[2]	QA (C23CHO, C16OH)		GlcA, Gal	Fuc(Ac), Rha, xyl; Araf
New vaccaria saponin 1[3]	QA (C23CHO, C16OH)		GlcA, Gal, Xyl	Fuc(Ac), Rha, xyl; Araf
Segetoside I[4]	QA (C23CHO, C16OH)		GlcA, Gal	Fuc(Ac), Rha, xyl; Araf(Ac)
New vaccaria saponin 2[3]	QA (C23CHO, C16OH)		GlcA, Gal, Xyl	Fuc(Ac), Rha, xyl; Araf(Ac)
Segetoside B[5]	QA (C23CHO, C16OH)		GlcA (Ac), Gal	Fuc(Ac), Rha, xyl; Araf(Ac)
vaccaroside F [1]	C23-OH, C16-OH		GlcA, Gal	Fuc(Ac), Rha, xyl; Araf
vaccaroside H[1]	C23-OH, C16-H		GlcA, Gal	Fuc(Ac), Rha, xyl; Araf
vaccaroside G[1]	GN (C23CHO, C16-H)		GlcA, Gal	Fuc(Ac), Rha, xyl; Araf
New vaccaria saponin 3[3]	GN (C23CHO, C16-H)		GlcA, Gal, Xyl	Fuc(Ac), Rha, xyl; Araf
Segetoside H[2]	GN (C23CHO, C16-H)		GlcA, Gal	Fuc(Ac), Rha, xyl; Araf(Ac)
New vaccaria saponin 4[3]	GN (C23CHO, C16-H)		GlcA, Gal, Xyl	Fuc(Ac), Rha, xyl; Araf(Ac)
Segetoside G[6]	GN (C23CHO, C16-H)		GlcA(CH3CH2CH2CH2), Gal	Fuc(Ac), Rha, xyl; Araf
segetoside C[7]	GA (C23COOH, C16-H)		H	G,G',G",G(Ac)
vaccaroid B[8]	GA (C23COOH, C16-H)		H	G,G',G",G, Acyl chain
vaccaroside C[9]	GA (C23COOH, C16-H)	Glc	H	G,G',G",G
Segetoside K[6]	C23COOH, C16-OH		H	G,G',G",G
Segetoside D [10]	C23H, C16-OH		GlcA(Ac), Gal	Fuc(Ac), Rha, xyl; Araf(Ac)
Segetoside E[7]	C23H, C16-OH		GlcA(CH3CH2CH2CH2), Gal	Fuc(Ac), Rha, xyl; Araf(Ac)

Supplementary Table 2.
List of accession numbers for Fig. 3a, Suppl. Fig. 12, and Suppl. Fig. 16

Gene name	accession ID
BfCYP716Y1-C16	KC963423
MtCYP716A12-C28	FN995113
PgCYP716A52v2-C28	JX036032
MlCYP716A75-C16	KF318733
MlCYP87D16-C16	KF318735
MtCYP72A68-C23	DQ335782
KsCYP72A397-C23	KT150517
BvCYP72A552-C23	MH252571
CaCYP714E19-C23	KT004520
IaCYP714E88-C23	MZ508437
CslG1 ARATH	NP_194132.3
CslG2 ARATH	NP_567692.2
CslG3 ARATH	NP_194130.3
Qs scaffold 2074300	QS_2074300 (1kp)
SO scaffold 2013703	SO_2013703 (1kp)
SO scaffold 2072976	SO_2013976 (1kp)
CslE1 ORYSJ	Q651X7
CslE6 ORYSJ	Q651X6
CslE2 ORYSJ	Q0DXZ1
CslE1 ARATH	Q8VZK9
CslB6 ARATH	O23386.2
CslB1 ARATH	O80898
CslH1 ORYSJ	Q339N5
CslF2 ORYSJ	Q84S11
CslD2 ARATH	Q9LFL0.1
CslD3 ORYSJ	Q7EZW6
CslC4 ARATH	Q9LJP4.1
CslA1 ARATH	Q84W54.1
CslA2 ARATH	Q9FNi7.1
CesA4 ORYSJ	Q5JN63
CESA1 ARATH	O48946
CESA7 ARATH	Q9SWW6
SOAP5	XP_021842158.1
PgUGT74AE2_Ginsenoside_CK-GlcT	A0A0A6ZFR4
SvUGT74M1_Gypsogenic_acid-C28-GlcT	ABK76266
SoUGT74BB2_Medicagenic_acid_3-GlcA-28-FucT	KNA07536.1
CitLGT_Limonoid-GlcT	BAA93039
SoUGT79K1_Yossoside_I-RhaT	KNA23861.1
SoUGT79L2_Yossoside_II-GlcT	KNA13786.1
GmUGT91H4_Soyasaponin_III-RhaT	BAI99585
GmUGT73P2_Soyasapogenol_B-GlcA-GalT	BAI99584
SoUGT73BS1_Yossoside_III-XylT	KNA07606.1
GuUGAT_Glycyrrhetinic-acid-3-GAT	ANJ03631.1
CsUGT73AM3_Cucurbitacin_3-O-glucopyranoside-GlcT	KGN59015

References

1. Jia, Z., Koike, K., Kudo, M., Li, H. & Nikaido, T. Triterpenoid saponins and sapogenins from Vaccaria segetalis. *Phytochemistry* **48**, 529–536 (1998).

2. Ma, C.-H. *et al.* Cytotoxic triterpenoid saponins from Vaccaria segetalis. *J. Asian Nat. Prod. Res.* **10**, 177–184 (2008).

3. Balsevich, J. J., Bishop, G. G. & Ramirez-Erosa, I. Analysis of bisdesmosidic saponins in Saponaria vaccaria L. by HPLC-PAD-MS: identification of new quillaic acid and gypsogenin 3-O-trisaccharides. *Phytochem. Anal.* **17**, 414–423 (2006).

4. Firempong, C. K. *et al.* Segetoside I, a plant-derived bisdesmosidic saponin, induces apoptosis in human hepatoma cells in vitro and inhibits tumor growth in vivo. *Pharmacol. Res.* **110**, 101–110 (2016).

5. Sang, S.-M. *et al.* A new triterpenoid saponin with inhibition of luteal cell from the seeds of Vaccaria segetalis. *J. Asian Nat. Prod. Res.* **4**, 297–301 (2002).

6. Sang, S. M., Lao, A. N., Chen, Z. L., Uzawa, J. & Fujimoto, Y. Three new triterpenoid saponins from the seeds of Vaccaria segetalis. *J. Asian Nat. Prod. Res.* **2**, 187–193 (2000).

7. Sang, S. *et al.* Triterpenoid saponins from Vaccaria segetalis. *J. Asian Nat. Prod. Res.* **1**, 199–205 (1999).

8. Yun, Y. S. *et al.* Triterpenoid saponin from Vaccaria segetalis. *Phytochemistry* **47**, 143–144 (1998).

9. Koike, K., Jia, Z. & Nikaido, T. Triterpenoid saponins from Vaccaria segetalis. *Phytochemistry* **47**, 1343–1349 (1998).

10. Tian, M. *et al.* Vaccaria segetalis: A Review of Ethnomedicinal, Phytochemical, Pharmacological, and Toxicological Findings. *Front. Chem.* **9**, 666280 (2021).
