# Supplementary material for: Deciphering triterpenoid saponin biosynthesis by leveraging transcriptome response to methyl jasmonate elicitation in Saponaria vaccaria
Source: Nat Commun. 2023 Nov 4;14:7101. doi: 10.1038/s41467-023-42877-0 (PMC10625584; doi:10.1038/s41467-023-42877-0)
Supplement: Supplementary file 3 — Description of Additional Supplementary Files [file 41467_2023_42877_MOESM3_ESM.pdf]

Description of Additional Supplementary Files

Supplementary Data 1

SvβAS co-induced genes and annotation.

Supplementary Data 2

Tab 1: List of primers and sequence of characterized genes.

Tab 2: Coding sequence of *S. vaccaria* genes that were characterized or used to construct the phylogenetic trees.

Supplementary Data 3

Sequences of proteins used to construct the tree in Supplementary Figure 19
