# Supplementary material for: Deciphering triterpenoid saponin biosynthesis by leveraging transcriptome response to methyl jasmonate elicitation in Saponaria vaccaria
Source: Nat Commun. 2023 Nov 4;14:7101. doi: 10.1038/s41467-023-42877-0 (PMC10625584; doi:10.1038/s41467-023-42877-0)
Supplement: Supplementary file 6 — Supplementary Data 3 [file 41467_2023_42877_MOESM6_ESM.pdf]

Amino acid sequences of enzymes in Supplementary Figure 19

>S.lycopersicum_GAME25

MANKLRLEGKVAIITGAASGIGEASARLFVEHGARVVVADIQDELGQKVVDSIGSDKAS

YRHCDVTDEKQVEETVAYAVEKYGTLDIMFSNVGTLNFCSVLDMDVLAFDETMAINVRG

SALAVKHAAKVMVDKKIRGSIICNASLEGILAGAASLAYIASKHAVVGIIKAAARELGP

HGIRVNGVSPYGIATPLVTKAYGLDAALLEEAIYGNGHLKGVKLSTMHVAQSALFLASD

ESAYTSGQNLAVDGGLSSILKLQ

>S.pennellii_GAME25

MANKLRLEGKVAIITGAASGIGEASARLFVEHGARVVVADIQDELGQKVVDSIGADKAS

YRHCDVTDEKQVEETVAYAVEKYGTLDIMFSNVGTLNFCSVLDMDVMAFDETMAINVRG

SALAVKHAAKVMVDKKIRGSIICNASLEGILAGAASLAYIASKHAVVGIIKAAARELGP

HGIRVNGVSPYGIATPLVCKAYGLDAALLEEAIYGNGHLKGVKLSTMHVAQSALFLASD

ESAYTSGQNLAVDGGLSSILKLQ

>S.tuberosum_GAME25

MANKLRLEGKVAIITGAASGIGEASARLFAEHGARIVVADIQDELGLKVVESIGADKAS

YRHCDVTDEKQVEDTVAYTVEKYGTLDIMFSNVGTLNFCSVLDMDVMVFDKTMAINARG

SALAVKHAARFMVDKKIRGSIICNASLDGIVAGATSLAYIASKHAVVGIVKAAARDLGP

YGIRVNGVSPYGIATPLVCKAYGLDAGPLEAAIYGNGNLKGVRLSTMHVAQSALFLASD

ESAYTSGQNLAVDGGLSSILKVQ

> S.lycopersicum_3βHSD

MASKLRLEGKVAIITGGASGIGEASARLFVQHGARVVVADIQDELGLQVVQSIGIHKAT

YRHCDVTDEKQVEDTVAYAVQKYATLDIMFSNVGTLNFCSVLDMDMTAFDETMTVNVRG

SALAVKHAARVMVDKKIRGSIICNVSLEGILAGAASLAYIASKHAVVGIVKAAARELGP

YGIRVNGVSPYGIATPLVCKAYGLDAAPLEAAINGNANLKGVTLSTMHVAQSALFLASD

ESAYTSGQNLAVDGGLSSILKLQ

> S.pennellii_3βHSD

MASKLRLEGKVAIITGGASGIGEASARLFVQHGARVVVADIQDELGLQVVQSIGIHKAT

YRHCDVTDEKQVEDTVAYAVQKYATLDVMFSNVGTLNFCSVLDMDMTAFDETMTVNVRG

SALAVKHAARVMVDKKIRGSIICNVSLEGILAGAASLAYIASKHAVVGIVKAAARELGP

YGIRVNGVSPYGIATPLVCKAYGLDAAPLEAAINGNANLKGVTLSTMHVAQSALFLASD

ESAYTSGQNLAVDGGLSSILKLQ

>C.annuum_3βHSD

LEGKVAVITGAASGIGEASARLFVEHGARVVIADIQDELGLQIAASIGTDKASYIHCDV

TDEKQVEEAVAYAVENSVLDLDVKAFDETMVINARGSAVAVKHAARVMVEKKIRGSIIC

TASLEGILAGAASLAYVSSKHAVVGLVKAAARELGVHGIRVNGVSPYGIATPLVCKAYG

LDAGPLETAIYGNAHLKGVTLSTMHVAQAALFLASDESAYISGQNLAVDGGLSSILKLE

>N.benthamiana_3βHSD

MANKLRLEGKVAVITGGASGIGEATARLFVEHGARVVIADIQDELGLQVVASIGTDKAS

YRHCDVTDENKVEETVAYAVEKYGTLDIMFSNVGTLNFCSVLDIDVTAFDKTMALNVRG

TALAVKHAARVMVAKQVKGSIICNASIEAILAGAASLAYVASKHAVVGIVKAAARELGL

HGIRVNGVSPYGIATPLVCKAYGCEDAASLEAGISVNAHLKGVTLSTEHIAQAALFLAS

DESAYISGHNLAVDGGLTSMLKLSY

>S.melongena_ADH1

MANKLKLEGKVAVITGGASGIGEESARLFVEHGARVVIADIQDDLGLEVVTSIGADKAC

YRHCDVSEEKQVKETVAYAVEKYGTLDIMFSNAGTLGTLGSVLEMDMTAFDMTMAVNMR

GSALAVKHAARVMVANKIRGSIICTASVEAILAGAAPLAYVASKHAILGVMKAAARELG

QYGIRVNCVSPYGIATPLVCKAYHSDAGSLEASIYERAHLKGITLSTKHIANASLFLAS

DESAYVSGHNLAVDGALSSIMS

>C.annuum_ADH1

MIFFFCGTRLEGKIAIITGAASGIGEASARLFVEHGAHVIIADIQDELGLQVVSSIGTD

KACYRHCDVTDEKQVEETVAYAVEKYGTLDIMFSNAGMLGTFGSLLDMDVKEFDLTIAV

NTRGAALAVKHAARVMVAKNIRGSIICTASVESILAGAAPLAYIASKHGILGVVKAAAR

ELGKNGIRVNCVSPFGIATPMVCKSYGAEASYIETSVGGHANLKGVSLTTKHIAEAALF

LASEESAYISGQNLAVDGGLSAIMRLD

>S.melongena_ADH2

MANKLRLEGKVAVITGGASGIGEASARLFVEHGARVVIADIQDELSLQVVSSIGGDKAC

YRRCDVSDEKQVEETVAYAVEKYGTLDIMFSNAGILGSFGSLLEMDMTAFDRIMAVNTR

GAALAVKHAARVMVANKIRGSIICTASVEAILAGEASLAYIASKHAILGVVKAAARDLG

QYGIRVNCVSPYGIATPMVCKSIGADAATIEARICGNANLKGVSLNTKHIAEAALFLGS

DESAYVSAHNLAVDGGLSSIMKLN

>S.tuberosum_ADH1

MANKLRLEGKVAVITGGASGIGEATARLFVEHGAHVVIADIQDELALQVVSSIGSDNVC

YRRCDVTDEKQVDETVAFAVQKYGTLDIMFSNAGILGSSGSLLEMDMAVFDRTMAVNTR

GAALAVKHAAKVMVAKKIRGSIICTASVESILAGAASLAYIASKHAVLGVVKAAARELG

QHGIRVNCVSPFGVATPMVCKSFGADAAAMEATIRGNANLKGVSLTTMHIAEAALFLAS

DESAYISAHNLAIDGGLSSIMKINVN

>S.lycopersicum_ADH1

MANKLRLEGKVAVITGGASGIGEAAARLFVEHGARVVIADIQDELALQVASSIGSDNVC

YQRCDVSDEKQVNETVAFAVEKYGTLDIMFSNAGILNPFESILEMDMTVFDRTIAVNAR

GAALAVKHAARVMVANKIRGSIICTASVESILAGAAPLAYIASKHAVLGVVKAAARELG

QHGIRVNCVSPFGIATPMVCKSFGADAAAIEAKICGNANLKGVSLTTMHIAEAALFLAS

DESAYISAQNLAVDGGLSSMMKLM

>S.pennellii_ADH1

MANKLRLEGKVAVITGGASGIGEAAARLFVEHGARVVIADIQDELALQVASSIGSVNVC

CRRCDVSDEKQVNETVAFAVEKYGTLDIMFSNAGILNPFESILEMDMTVFDRTIAVNAR

GAALAVKHAARVMVANKIRGSIICTASVESILAGAAPLAYIASKHAVLGVVKAAARELG

QHGIRVNCVSPFGIATPMVCKSFGADAAAIEAKICGNANLKGVSLTTMHIAEAALFLAS

DESAYISAQNLAVDGGLSSMMKLM

>N.benthamiana_ADH1

MANKRRLEGKVAVITGAASGIGEATARLFVEHGARVVIADIQDELGHQVVASIGTDKAS

YRHCDVTDEKQVEDTVVYTVEKYGTLDIMFSNAGTIGTLGSILDMDMTVFDRTMAINAR

GSALAVKHAARVMVTKKIQGSIICTASLEATLAGAAPLAYVTSKHAILGVVKAAARELG

QHGIRVNCVSPYGIATPMVCKTFGGDAAPIEASISGNANLKGITLSTKHIAEAALFLAS

DESAYVSAHNLAVDGGLSSIMKLD

>N.benthamiana_ADH2

MANKLRLEGKVAVITGAASGIGEATARLFVEHGARVVIADIQDELGHQVVASIGTDKAS

YRHCDVTDEKQVEDTVVYAVEKYGTLDIMFSNAGTIGTLSSILDMDMTVFDRTMAINAR

GSALAVKHAARIMVTKKIQGSIICTASLEAILAGAAPLAYVASKHAILGVVKAAARELG

QHGIRVNCVSPYGIATPMVCKTFGGDAAPIEASISGNANLKGITLSTKHIAEAALFLAS

DESAY

>A.thaliana_ADH

MSGKRLDGKIVIITGGASGIGAESVRLFTEHGARVVIVDVQDELGQNVAVSIGEDKASY

YHCDVTNETEVENAVKFTVEKYGKLDVLFSNAGVIEPFVSILDLNLNELDRTIAINLRG

TAAFIKHAARAMVEKGIRGSIVCTTSVAAEIAGTAPHGYTTSKHGLLGLIKSASGGLGK

YGIRVNGVAPFGVATPLVCNGFKMEPNVVEQNTSASANLKGIVLKARHVAEAALFLASD

ESAYVSGQNLAVDGGYSVVKP

>M.truncatula_ADH

MSRKRLEGKVAIVTGGASGIGAETAKTFVENGAFVVIADINDELGHQVATSIGLDKVSY

HHCDVRDEKQVEETVAFALEKYGTLDIMFSNAGIEGGMSSSILEFDLNEFDNTMAINVR

GSLAAIKHAARFMVERKIRGSIICTASVAASVAGNRGHDYVTSKHGLLGLVRSTCGELG

AYGIRVNSISPYGVATPLACRALNMEMSKVEANMKDSANLKGITLKATHIAEAALFLAS

EESAYISGHNLVVDGGFSVINSCVPTTIKK

>C.annuum_ADH2

MAVVMQKLKGKVAIVTGGASGIGEATVRLFAEHGARAVVIADIQDEKGRAVAESIPLQV

CSYVHCDVSDENQVKGLVDWTVKKYGQLDIMFSNAGTVGNSGQKVLDLDLSEFDRVIRV

NARGMAACVKHAARAMVEQGGRGSIICTGSVGASKGAAWRTDYTMSKHAVLGLVTSASR

QLGKYGIRVNSISPSAVMTPLMSSAEAETSMKVLKMYGPLTSLKGITLTVKHLADAVLF

LASDDSAFVNGHDLLVDGGLLHLPDPMSSL

>S.tuberosum_ADH2

MAEVTQKLKGKVAIVTGGASGIGEATARLFAQHGARAVVIADIQDGKGRAVAVSIPSQI

CSYVQCDVSDENQVKAMVDWTVQKYGQLDIMFSNAGVVGNSGQKVLDLDLSEFDRVMNV

NARGMAACVKHAARAMVDKRVRGSIICTGSIGASRGGAWRTDYIMSKHAVLGLVRSACR

QLGEYGIRVNSISPSAVMTPLMISAEPEVSMKSLKRYGPQTSLKGITLTVKHLAEAALF

LASDDSAFSSRSNEFIVKQREQPNLSLFFF

>S.melongena_ADH3

MSAITQKLNGKVAIVTGGASGIGEATVRLFAQHGARAVVIADIQDEKGRAVAQSIPSQI

CIYVKCDVSDENQVKSMVDWTVQQYGQLDIMFSNAGTVGNSGQKILDLDLSEFDRVMNV

NARGMAACVKHAARAMVEKRVRGSIICTGSIAASRAGAWRTDYAMSKHAVLGLMRSASR

QLGEYGIRVNSISPSAVMTPLMISAEAEASMRVLKMYGSVTSLKGITLTVKHLADAVLF

LASDDSVFVSGHDLAVDGGLISLPDPMSSL

>O.sativa_MIS2

MFTAMHRILSRGRRTPAASSSSVTAFATASDSQRLAGKVAVITGGASGIGRATAEEFVR

NGAKVILADVQDDLGHAVAAELGADAASYARCDVTDEAQVAAAVDLAVARHGRLDVVFN

NAGIPGDLTPTPVGALDLADFDRVMAVNTRAVVAGVKHAARVMVPRRRGSIICTASTAG

VIGGVAVPHYSVSKAAVLGLVRAVAGEMARSGVRVNAISPNYIWTPMAAVAFARWYPSR

SADDHRRIVENDINEMDGVTLEAEDVARAAVFLASDEAKYVNGHNLVVDGGYTVGKVPN

MPVPDGH

>O.sativa_MIS3

MFRAAQLLLRETNRALGAATSPAGFVSGFSTASNSAQRLAGKVAVITGGASGIGKATAK

EFIENGAKVIMADVQDDLGHSTAAELGPDASYTRCDVTDEAQVAAAVDLAVKRHGHLDI

LYNNAGVMGAMPQDDMASVDLANFDRMMAINARAALVGIKHAARVMSPRRSGVILCTAS

DTGVMPMPNIALYAVSKATTIAIVRAAAEPLSRHGLRVNAISPHGTRTPMAMHVLSQMY

PGVSKDDLEKMADAAMDAGEVMEPKYVARAALYLASDEAKYVNGHNLVVDGGFTSHKGS

DTRLN

>A.thaliana_ABA2

MSTNTESSSYSSLPSQRLLGKVALITGGATGIGESIVRLFHKHGAKVCIVDLQDDLGGE

VCKSLLRGESKETAFFIHGDVRVEDDISNAVDFAVKNFGTLDILINNAGLCGAPCPDIR

NYSLSEFEMTFDVNVKGAFLSMKHAARVMIPEKKGSIVSLCSVGGVVGGVGPHSYVGSK

HAVLGLTRSVAAELGQHGIRVNCVSPYAVATKLALAHLPEEERTEDAFVGFRNFAAANA

NLKGVELTVDDVANAVLFLASDDSRYISGDNLMIDGGFTCTNHSFKVFR

>O.sativa_MS3

MAGSSYGDVHESARKLVGKVALITGGASGIGECTARLFVKHGAQVVVADIQDEAGARLC

AELGSATASYVRCDVTSEDDVAAAVDHAVARYGKLDVMFNNAGIGGAACHSILESTKAD

FDRVLAVNLTGPFLGTKHAARVMVAAGRGGCIIGTASLASAVAGTASHAYTCAKRALVG

LTENAAAELGRHGIRVNCVSPAAAATPLATGYVGLEGEAFEAAMEAVANLKGVRLRVED

IAAAVLFLASDDARYVSGHNLLIDGGCSIVNPSFGIFKD

>O.sativa_MS1

MAAGSSHVSADARKLVGKVAVITGGASGIGACTARLFVKHGARVVVADIQDELGASLVA

ELGPDASSYVHCDVTNEGDVAAAVDHAVARFGKLDVMFNNAGVSGPPCFRMSECTKEDF

ERVLAVNLVGPFLGTKHAARVMAPARRGSIISTASLSSSVSGAASHAYTTSKHALVGFT

ENAAGELGRHGIRVNCVSPAGVATPLARAAMGMDDEAIEAIMANSANLKGAGALKADDI

AAAALFLASDDGRYVSGQNLRVDGGLSVVNSSFGFFRD

>O.sativa_MS2

MAGSSHVSADARKLVGKVAVITGGASGIGACTARLFVKHGARVVVADIQDELGASLVAE

LGPDASSYVHCDVTNEGDVAAAVDHAVATFGKLDVMFNNAGVTGPPCFRITESTKEDFE

RVLAVNLIGPFLGTKHAARVMAPARRGSIISTASLSSSVSGTASHAYTTSKRALVGFTE

NAAGELGRHGIRVNCVSPAAVATPLARAAMGMDMDDETIEAIMEKSANLKGVGLKVDDI

AAAALFLASDDGRYVSGQNLRVDGGVSVVNSSFGFFRD

>A.thaliana_11/17-βHSD

MELINDFLNLTAPFFTFFGLCFFLPPFYFFKFLQSIFSTIFSENLYGKVVLITGASSGI

GEQLAYEYACRGACLALTARRKNRLEEVAEIARELGSPNVVTVHADVSKPDDCRRIVDD

TITHFGRLDHLVNNAGMTQISMFENIEDITRTKAVLDTNFWGSVYTTRAALPYLRQSNG

KIVAMSSSAAWLTAPRMSFYNASKAALLSFFETMRIELGGDVHITIVTPGYIESELTQG

KYFSGEGELIVNQDMRDVQVGPFPVASASGCAKSIVNGVCRKQRYVTEPSWFKVTYLWK

VLCPELIEWGCRLLYMTGTGMSEDTALNKRIMDIPGVRSTLYPESIRTPEIKSD

>A.thaliana_TR

METDKRWSLAGKTALVTGGTRGIGRAVVEELAKFGAKVHTCSRNQEELNACLNDWKANG

LVVSGSVCDASVRDQREKLIQEASSAFSGKLNILINNVGTNVRKPTVEYSSEEYAKIMS

TNLESAFHLSQIAHPLLKASGVGSIVFISSVAGLVHLSSGSIYGATKGALNQLTRNLAC

EWASDNIRTNCVAPWYIKTSLVETLLEKKEFVEAVVSRTPLGRVGEPEEVSSLVAFLCL

PASSYITGQVISVDGGFTVNGFSYAMKP

>Z.mays_LCR

MSTGGRKMRTACVTGGNGYIASALIKVLLEKGYAVKTTVRNPDDMEKNSHLKDLQALGS

LEVFRADLDEDGSFDDAVAGCDYAFLVAAPVNLHTKNPEEEMIEPAVRGTLNVMRSCVK

AGTVRRVVLTSSAAAVTTRPQLQGDGHVLDEESWSDVEYLRAHKPAGPWGYPVSKVLLE

KEASRFAEEHGIGLVTVCPGLTVGAAPAPTARTSVPNCLSLLSGDEAAFAVLDAIESAT

GCLPLVHVDDVCRAELFAAEEGAAARRYVCCGLNTTVAELARFLADKYPQYGVKTNLLS

GERLEKPRVCLSSAKLVKEGFEFRYRTLDDIYDDMVEYGKALGILPDL

>A.thaliana_ACR

MDQTLTHTGSKKACVIGGTGNLASILIKHLLQSGYKVNTTVRDPENEKKIAHLRKLQEL

GDLKIFKADLTDEDSFESSFSGCEYIFHVATPINFKSEDPEKDMIKPAIQGVINVLKSC

LKSKSVKRVIYTSSAAAVSINNLSGTGIVMNEENWTDVEFLTEEKPFNWGYPISKVLAE

KTAWEFAKENKINLVTVIPALIAGNSLLSDPPSSLSLSMSFITGKEMHVTGLKEMQKLS

GSISFVHVDDLARAHLFLAEKETASGRYICCAYNTSVPEIADFLIQRYPKYNVLSEFEE

GLSIPKLTLSSQKLINEGFRFEYGINEMYDQMIEYFESKGLIKAK

>P.somniferum_NOS

MHGQKNISERYQKFKEMEGTGKIVCVTGGAGYLASWLIMRLLERGYSVRTTVRSDPKFR

EDVSHLKALPEATEKLQIFEADLENPESFDDAINGCVGVFLVAQGMNFAEEYTLEKIIK

TCVEGTLRILQSCLKSKTVKKVVYTSSADAAMMISNLKAVKEIDETIWSEVDNFISKPE

QVIPGLPSYVVSKVLTERACLKFSEEHGLDVVTILPPLVVGPFITPHPPPSVSIALSII

SGDVSMMLGVRLENAVHIDDVALAHIFVFECEKAKGRHICSSVDFPMHDLPKFISENYP

EFNVPTDLLKDIEEQEPVHLSSDKLLSMGFQFKYDFAEIFGDAIRCAKEKGFL

>A.thaliana_4-DFR

MVREEEEDDNNGGGGERKLPVADETVPSLLDGTGLVCVTGGTGFVASWLIMRLLQRGYS

VRATVRTNPEGNKKDISYLTELPFASERLQIFTADLNEPESFKPAIEGCKAVFHVAHPM

DPNSNETEETVTKRTVQGLMGILKSCLDAKTVKRFFYTSSAVTVFYSGKNGGGGGEVDE

SVWSDVEVFRNQKEKRVSSSYVVSKMAAETAALEFGGKNGLEVVTLVIPLVVGPFISPS

LPSSVFISLAMLFGNYKEKYLFDTYNMVHIDDVARAMILLLEKPVAKGRYICSSVEMKI

DEVFEFLSTKFPQFQLPSIDLKNYKVEKRMSLSSKKLRSEGFEFKYGAEEIFGGAIRSC

QARGFL

>H.sapiens_AKR

MDPKYQRVELNDGHFMPVLGFGTYAPPEVPRNRAVEVTKLAIEAGFRHIDSAYLYNNEE

QVGLAIRSKIADGSVKREDIFYTSKLWCTFFQPQMVQPALESSLKKLQLDYVDLYLLHF

PMALKPGETPLPKDENGKVIFDTVDLSATWEVMEKCKDAGLAKSIGVSNFNCRQLEMIL

NKPGLKYKPVCNQVECHPYLNQSKLLDFCKSKDIVLVAHSALGTQRHKLWVDPNSPVLL

EDPVLCALAKKHKRTPALIALRYQLQRGVVVLAKSYNEQRIRENIQVFEFQLTSEDMKV

LDGLNRNYRYVVMDFLMDHPDYPFSDEY

>M.musculus_AKR

MDSKQQTVRLSDGHFIPILGFGTYAPQEVPKSKATEATKIAIDAGFRHIDSASMYQNEK

EVGLAIRSKIADGTVKREDIFYTSKVWCTFHRPELVRVCLEQSLKQLQLDYVDLYLIHF

PMAMKPGENYLPKDENGKLIYDAVDICDTWEAMEKCKDAGLAKSIGVSNFNRRQLEKIL

KKPGLKYKPVCNQVECHPYLNQGKLLDFCRSKDIVLVAYSALGSHREKQWVDQSSPVLL

DNPVLGSMAKKYNRTPALIALRYQLQRGVVVLAKSFSEKRIKENMQVFEFQLTSEDMKV

LDDLNKNIRYISGSSFKDHPDFPFWDEY

>S.lycopersicum_AKR2

MAEATEMPYIELNTGFSIPAVGLGTWQSDPGVVGKAVETAIKMGYRHIDCAQIYKNEKE

IGEVLSRLFKDGVVKRRELFITSKLWNTNHAPEDVPVALDKTLQDLQLEYVDLYLIHWP

VSMKPGSVDFKPENLMPTNIPRIWEAMEKVYDSGKARVIGVSNFSTKKLEDLLQVARTP

PAVNQVECHPSWQQAKLRELCKSNNVHLSAYSPLGSPGTTWLKSDVLKQPAVISVAEKL

GKTPAQVCLRWGIQMGQSVLPKSTHEARIKENLDVLNWSIPDDLLAKFSEIPQARLLKG

ASFAHETHGQYRTLEELWDGEI

>S.lycopersicum_AKR1

MTMNLIKQMLVPNVNLNSGHKMPLIGMGTAPSLPEHDQLVSTLIDAIEIGYRHFDTAAV

YGSEEALGQAVVEAIQRGLIKSREQVFITSKLWCTETHRHLVLPALKRTLGRLKMDYLD

LYLIHLPVTMKKKVNSKDDEMRVDKEDIIPFDMRGTWEAMEECCRLGLAKSIGVSNFTC

TKISQILHYATILPAVNQVEMHVAWRQEKMLEFCKEKGIHVSAWSPLGANGLTPWGIHS

VMESPVLKDIAIHKRKSVAQVALRWVYEQGASVIVKSFNKERMKENLQILDWELSNEEI

AQIQEIPPCTGFNVDMVLVHPNGPYKSANQFWDGEI

>M.truncatula_AKR

MGSVEIPTKVLTNTSSQLKMPVVGMGSAPDFTCKKDTKDAIIEAIKQGYRHFDTAAAYG

SEQALGEALKEAIELGLVTRQDLFVTSKLWVTENHPHLVIPALQKSLKTLQLDYLDLYL

IHWPLSSQPGKFTFPIDVADLLPFDVKGVWESMEEGLKLGLTKAIGVSNFSVKKLENLL

SVATILPAVNQVEMNLAWQQKKLREFCNANGIVLTAFSPLRKGASRGPNEVMENDMLKE
IADAHGKSVAQISLRWLYEQGVTFVPKSYDKERMNQNLCIFDWSLTKEDHEKIDQIKQN
RLIPGPTKPGLNDLYDD

>P.somniferum_COR

MESNGVPMITLSSGIRMPALGMGTAETMVKGTEREKLAFLKAIEVGYRHFDTAAAYQTE
ECLGEAIAEALQLGLIKSRDELFITSKLWCADAHADLVLPALQNSLRNLKLDYLDLYLI
HHPVSLKPGKFVNEIPKDHILPMDYKSVWAAMEECQTLGFTRAIGVCNFSCKRLQELME
TANSPPVVNQVEMSPTLHQKNLREYCKANNIMITAHSVLGAVGAAWGTNAVMHSKVLHQ
IAVARGKSVAQVSMRWVYQQGASLVVKSFNEARMKENLKIFDWELTAEDMEKISEIPQS
RTSSAAFLLSPTGPFKTEEEFWDEKD

>A.thaliana_AKR

MSALTFPIGSVHHLMPVLALGTAASPPPEPIVLKRTVLEAIKLGYRHFDTSPRYQTEEP
LGEALAEAVSLGLIQSRSELFVTSKLWCADAHGGLVVPAIQRSLETLKLDYLDLYLIHW
PVSSKPGKYKFPIEEDDFLPMDYETVWSEMEECQRLGVAKCIGVSNFSCKKLQHILSIA
KIPPSVNQVEMSPVWQQRKLRELCKSKGIVVTAYSVLGSRGAFWGTHKIMESDVLKEIA
EAKGKTVAQVSMRWAYEEGVSMVVKSFRKDRLEENLKIFDWSLTEEEKQRISTEISQSR
IVDGEVYISEKGPIKSVTEMWDGEI

>D.lanata_3βHSD

MSSKPRLEGKVAIITGAASGIGEETARLFVEHGASVVVADVQDELGRQVVASVNSDDKI
SYYHCDVRDEKQVAATVRYAVEKYGRLDIMLSNAGVFGALMTNVIDLDMVDFENVLATN
VRGVANTIKHAARAMVEGKVKGSIICTASVSASLGGMGPPAYTASKHAVLGLVKGACAE
LGVHGIRVNSVAPYGVATPMPCSAYGMTPSQMEEANNSRANLKGVVLKAKHVAEAALFL
ASDESAYVSGQNLAVDGGFTVVR

>NAD(P)-binding Rossmann-fold superfamily protein [Arabidopsis thaliana]

MAEETPRLFNGFCRYAVVTGANRGIGFEICRQLASEGIRVVLTSRDENRGLEAVETLKKELEISDQSLLF
HQLDVADPASITSLAEFVKTQFGKLDILVNNAGIGGIITDAEALRAGAGKEGFKWDEIITETYELTEECI
KINYYGPKRMCEAFIPLLKLSDSPRIVNVSSSMGQLKNVLNEWAKGILSDAENLTEERIDQVINQLLNDF
KEGTVKEKNWAKFMSAYVVSKASLNGYTRVLAKKHPEFRVNAVCPGFVKTDMNFKTGVLSVEEGASSPVR
LALLPHQETPSGCFFSRKQVSEF

>Mentha x piperita NMD

MGDEVVVDHAATKRYAVVTGANKGIGFEICKQLASKGITVILASRDEKRGIEARERLIKE

LGSEFGDYVVSQQLDVADPASVAALVDFIKTKFGSLDILVNNAGLNGTYMEGDASVLNDY

VEAEFKTFQSGAAKTEPYHPKATGRLVETVEHAKECIETNYYGSKRVTEALIPLLQQSDS

PRIVNVSSTLSSLVFQTNEWAKGVFSSEEGLTEEKLEEVLAEFLKDFIDGKQQEKQWPPH

FSAYKVSKAALNAYTRIIAKKYPSFRINAVCPGYTKTDLSYGHGQFTDAEAAEAPVKLAL

LPQGGPSGCFFFRDEAFCLY

>Spinacia oleracea NMD

MAEQSNFLAEKRYAVVTGANKGIGLEICRQLASQGVIVLITSRDGKKGLEALNDLIKSGISSDNLHYHQL

DVTDPMSITALAGFINSKFGKLDILVNNAGIGGFIIDYDAIKAIGFRNINYDEMMTQTYELAKECLETNY

YGVKRTTEALLPQLELSDSPRIINVSSSTGMLKNIPNERIRGVLGDAENLTEEKVEAILNELLTDFKDGS

FKEKEWPSRMAAYTLSKAALNAYARILAKKYPSIIISCVCPGVTKTDMNGNLGQLTVEEGAASPVRVALM

PHGSPSGLFYARSEVSSYE

>P.bracteatum SR

MPETCPNTVTKMRCAVVTGGNKGIGFEICKQLSSSGIMVVLTCRDVTRGLEAVEKLKNS

NHENVVFHQLDVTDPITTMSSLADFIKARFGKLDILVNNAGVAGFSVDADRFKAMISDI

GEDSEEVVKIYEKPEAQELMSETYELAEECLKINYYGVKSVTEVLLPLLQLSDSPRIVN

VSSSTGSLKYVSNETALEILGDGDALTEERIDMVVNMLLKDFKENLIETNGWPSFGAAY

TTSKACLNAYTRVLAKKIPKFQVNCVCPGLVKTEMNYGIGNYTADEGAKHVVRIALFPD

DGPSGFFYDCSELSAF

>Z.mays SDR

MDAAAAAASPTSKRIALVTGGNKGIGLETCRQLASRGVRVVLTARNEARGLEAVERVRC

ARGDAEVYFHQLDVTDPCSAARLADFVRDQFGRLDILINNAGISGVHRDPVLSAAVKDK

VDGMDVNQRVEWMKENSKETYEEAVQCMKTNYYGAKLVTEALLPLLQLSSSGRIVNVSS

GFGLLRNFNSEDLRKEFEDIDNLTESRLEELMDKFLEDFKANLVEEHGWPTGGSSAYKV

VKAALNAYTRILAKKYPTLRINCLTPGYVKTDISMHMGVLTLEEGARNPVKVALLPDDG

PTGAYFDLNGEASFV

>QsFucSyn

MAEATQRYAVVTGSNKGIGFGICKQLASKGIKVVLTARDEKRGLEAVEKL

KEISLAGHVVFHQLDVSDPASVTSLEDFIKTQFGKLDILVNNAGITGTTV

DADALAASGFGTGGERKPIDWSKLVIQTYESVEKAFNTNYYGGKRMTEAL

IPLLQLSDSPRIVNVSSAMGQLENIPSGWAKEVLTDVDNLTEGKLDEVST

QFLKDFKEGSLETKGWPSLMSSYIVSKAVLNAYTRILAKKYPAFCINCVD

PGYVKTDINHHTGQLSVDEGAESPVRLALLPNGGPSGVFFSRTEEAPF

>SKNL_scaffold_2072503

AQKRYAVVTGANKGLGLEICGQLASQGVTVLLTSRDEKRGLEAIEELKKSGINSENLEYH

QLDVTKPASFASLADFIKAKFGKLDILVNNAGISGVIVDYAALMEAIRRRGAEINYDGVM

KQTYELAEECLQTNYYGVKRTINALLPLLQFSDSPRIVNVSSDVGLLKKIPGERIREALG

DVEKLTEESVDGILDEFLRDFKEGKIAEKGWPTFKSAYSISKAALNSYTRVLARNYPSII

INCVCPGVVKTDINLKMGHLTVEEGAASPVRLALMPLGSPSGLFYTRNEVTPF
