# Supplementary material for: Deciphering triterpenoid saponin biosynthesis by leveraging transcriptome response to methyl jasmonate elicitation in Saponaria vaccaria
Source: Nat Commun. 2023 Nov 4;14:7101. doi: 10.1038/s41467-023-42877-0 (PMC10625584; doi:10.1038/s41467-023-42877-0)
Supplement: Supplementary file 7 — Reporting Summary [file 41467_2023_42877_MOESM7_ESM.pdf]

nature portfolio

Corresponding author(s): Henrik V. Scheller

Last updated by author(s): Oct 15, 2023

Reporting Summary

Nature Portfolio wishes to improve the reproducibility of the work that we publish. This form provides structure for consistency and transparency in reporting. For further information on Nature Portfolio policies, see our Editorial Policies and the Editorial Policy Checklist.

Statistics

For all statistical analyses, confirm that the following items are present in the figure legend, table legend, main text, or Methods section.

n/a	Confirmed	
☐	☒	The exact sample size (*n*) for each experimental group/condition, given as a discrete number and unit of measurement
☐	☒	A statement on whether measurements were taken from distinct samples or whether the same sample was measured repeatedly
☐	☒	The statistical test(s) used AND whether they are one- or two-sided<br>*Only common tests should be described solely by name; describe more complex techniques in the Methods section.*
☒	☐	A description of all covariates tested
☐	☒	A description of any assumptions or corrections, such as tests of normality and adjustment for multiple comparisons
☐	☒	A full description of the statistical parameters including central tendency (e.g. means) or other basic estimates (e.g. regression coefficient) AND variation (e.g. standard deviation) or associated estimates of uncertainty (e.g. confidence intervals)
☐	☒	For null hypothesis testing, the test statistic (e.g. *F*, *t*, *r*) with confidence intervals, effect sizes, degrees of freedom and *P* value noted<br>*Give P values as exact values whenever suitable.*
☒	☐	For Bayesian analysis, information on the choice of priors and Markov chain Monte Carlo settings
☒	☐	For hierarchical and complex designs, identification of the appropriate level for tests and full reporting of outcomes
☒	☐	Estimates of effect sizes (e.g. Cohen's *d*, Pearson's *r*), indicating how they were calculated

Our web collection on statistics for biologists contains articles on many of the points above.

Software and code

Policy information about availability of computer code

Data collection	not applicable for our study; no software was used for data collection.
Data analysis	Microsoft Excel and GraphPad Prism softwares were used for statistical analysis. PacBio raw data were analyzed using Iso-Seq v3.2.2 (https://github.com/PacificBiosciences/IsoSeq_SA3nUP) bioinformatics pipeline. Nonredundant transcripts were assembled to reconstruct unique transcript models with Cogent v6.0.0 (https://github.com/Magdoll/Cogent). Nonredundant transcripts were mapped to UniTransModels using minimap2 v2.9. Nonredundant transcripts were further collapsed into unique transcript isoforms via cDNA Cupcake v 12.1.0 (https://github.com/Magdoll/cDNA_Cupcake). Alternative splicing events were detected with Astalavista (http://astalavista.sammeth.net/). Unique transcript isoforms were annotated with Trinotate (https://github.com/Trinotate/Trinotate.github.io/wiki). Raw Illumina reads were cleaned using BBDuk (https://jgi.doe.gov/data-and-tools/bbtools/bb-tools-user-guide/bbduk-guide/). Clean reads were mapped to S. vaccaria PacBio full-length unique transcript isoforms using Salmon v1.2.1. Differential expression analysis was performed using limma-voom. A GO enrichment analysis of differentially expressed genes was performed with the GOseq package. The differentially expressed genes were partitioned into gene clusters with the script "define_clusters_by_cutting_tree.pl" in Trinity (https://github.com/trinityrnaseq/trinityrnaseq/wiki/Trinity-Differential-Expression). Enzyme kinetics was calculated using GraphPad Prism software. LC-MS analysis was performed using Agilent OpenLab ChemStation software. LC-QTOF-MS data acquisition (Workstation B.08.00) and processing (Qualitative Analysis B.06.00) were performed via Agilent Technologies MassHunter software. Alignment of protein sequences were performed using ClustalX2.

For manuscripts utilizing custom algorithms or software that are central to the research but not yet described in published literature, software must be made available to editors and reviewers. We strongly encourage code deposition in a community repository (e.g. GitHub). See the Nature Portfolio guidelines for submitting code & software for further information.

Data

Policy information about availability of data

All manuscripts must include a data availability statement. This statement should provide the following information, where applicable:

- Accession codes, unique identifiers, or web links for publicly available datasets
- A description of any restrictions on data availability
- For clinical datasets or third party data, please ensure that the statement adheres to our policy

The unaligned CCS bam files of PacBio sequencing are deposited under NCBI BioProject ID PRJNA951399 [https://www.ncbi.nlm.nih.gov/bioproject/PRJNA951399]. Clean and trimmed RNASeq reads are deposited under NCBI BioProject ID PRJNA949801 [https://www.ncbi.nlm.nih.gov/bioproject/PRJNA949801]. An annotated list of transcripts with altered expression after MeJA treatment is provided in Supplementary Data 1. The sequences of genes characterized in this work can be found in Supplementary Data 2. Source data are provided with this paper.

Human research participants

Policy information about studies involving human research participants and Sex and Gender in Research.

Reporting on sex and gender	n/a
Population characteristics	n/a
Recruitment	n/a
Ethics oversight	n/a

Note that full information on the approval of the study protocol must also be provided in the manuscript.

Field-specific reporting

Please select the one below that is the best fit for your research. If you are not sure, read the appropriate sections before making your selection.

- [x] Life sciences
- [ ] Behavioural & social sciences
- [ ] Ecological, evolutionary & environmental sciences

For a reference copy of the document with all sections, see nature.com/documents/nr-reporting-summary-flat.pdf

Life sciences study design

All studies must disclose on these points even when the disclosure is negative.

Sample size	Statistical based sample size calculation was not applicable to our study. Details of technical/biological replicates related to various experiments are provided in the manuscript.
Data exclusions	No data was excluded in this study.
Replication	Details of technical/biological replicates used in various experiments are provided in methods section as well as in Main Figures and Supplementary Figures legends.
Randomization	Not applicable for our study.
Blinding	Not applicable for our study.

Reporting for specific materials, systems and methods

We require information from authors about some types of materials, experimental systems and methods used in many studies. Here, indicate whether each material, system or method listed is relevant to your study. If you are not sure if a list item applies to your research, read the appropriate section before selecting a response.

Materials & experimental systems

n/a	Involved in the study	
☒	☐	Antibodies
☒	☐	Eukaryotic cell lines
☒	☐	Palaeontology and archaeology
☒	☐	Animals and other organisms
☒	☐	Clinical data
☒	☐	Dual use research of concern

Methods

n/a	Involved in the study	
☒	☐	ChIP-seq
☒	☐	Flow cytometry
☒	☐	MRI-based neuroimaging
